# Understanding heterogeneity in Genesis diamond-like carbon film using SIMS analysis of implants

**DOI:** 10.1007/s10853-017-1267-3

**Published:** 2017-07-05

**Authors:** Amy J. G. Jurewicz, Don S. Burnett, Karen D. Rieck, Richard Hervig, Tom A. Friedmann, Peter Williams, Charles P. Daghlian, Roger Wiens

**Affiliations:** 1 Center for Meteorite Studies, School of Earth and Space Exploration, Arizona State University, PO. Box 876004, Tempe, AZ 85287-6004 USA; 2 Department of Geology and Planetary Sciences, California Institute of Technology, m/c 100-10, Pasadena, CA 91125 USA; 3 ISR-2 (Space and Remote Sensing), Los Alamos National Laboratory, m/s D-434, Los Alamos, NM 87545 USA; 4 School of Earth and Space Exploration, Arizona State University, Tempe, AZ 85287 USA; 5 Department of Nanostructure and Semiconductor Physics, Sandia National Laboratories, Albuquerque, NM 87185-1415 USA; 6 School of Molecular Science, Arizona State University, Tempe, AZ 85287 USA; 7 Electron Microscope Facility, Geisel School of Medicine, Dartmouth College, Hanover, NH 03755 USA; 8 ISR-1 (Space Science and Applications), Los Alamos National Laboratory, m/s D-466, Los Alamos, NM 87545 USA

## Abstract

An amorphous diamond-like carbon film deposited on silicon made at Sandia National Laboratory by pulsed laser deposition was one of several solar wind (SW) collectors used by the Genesis Mission (NASA Discovery Class Mission #5). The film was ~1 μm thick, amorphous, anhydrous, and had a high ratio of *sp* ^3^–*sp* ^2^ bonds (>50%). For 27 months of exposure to space at the first Lagrange point, the collectors were passively irradiated with SW (H fluence ~2 × 10^16^ ions cm^−2^; He fluence ~8 × 10^14^ ions cm^−2^). The radiation damage caused by the implanted H ions peaked at 12–14 nm below the surface of the film and that of He about 20–23 nm. To enable quantitative measurement of the SW fluences by secondary ion mass spectroscopy, minor isotopes of Mg (^25^Mg and ^26^Mg) were commercially implanted into flight-spare collectors at 75 keV and a fluence of 1 × 10^14^ ions cm^−2^. The shapes of analytical depth profiles, the rate at which the profiles were sputtered by a given beam current, and the intensity of ion yields are used to characterize the structure of the material in small areas (~200 × 200 ± 50 μm). Data were consistent with the hypothesis that minor structural changes in the film were induced by SW exposure.

**Electronic supplementary material:**

The online version of this article (doi:10.1007/s10853-017-1267-3) contains supplementary material, which is available to authorized users.

## Background

### The opportunity presented by the Genesis solar wind sample return mission

The Genesis spacecraft orbited the Lagrange (L1) point for 27 months, during which time panels holding a variety of materials faced directly toward the Sun to expose them to the solar wind (SW). The purpose of the Genesis Mission was to return a sample of SW in order to measure the composition of the minor SW ions accurately and precisely. The SW composition defines the composition of the outer portion of the Sun and, by extension, the composition of the solar nebula from which our solar system formed [1]. The SW is a flux of high-velocity ions consisting primarily of H with a significant amount of He. So, collecting the SW was equivalent to exposing collector materials to a long duration, low temperature, low current ion implant of, to first order, ~2 × 10^16^ ions cm^−2^ H at a distribution of energies. However, He is also a significant component of SW (He/H = 0.039), and trace amounts of the entire periodic table are present as well [2, 3].

One of the many types of collectors implanted at the L1 point and then returned to Earth for analysis was “Diamond on Silicon” (DoS), i.e., silicon substrates coated with amorphous, anhydrous, diamond-like carbon (DLC) films approximately 1 µm thick having a tetrahedral amorphous carbon structure [4]. Only the film portion of the DoS wafer collected the SW, and truly only the top half of the film was used; e.g., data from analyses (cf., “The Genesis SW sample” in Experimental Details) indicate that the SW H implant peaked at 12.0–14.0 nm and the He peaked at 20.0–23.0 nm. The Mg peak was about 20.0–26.0 nm below the DLC surface.

An important aside: the return of the Genesis samples to Earth was not nominal. The drogue parachute failed to open, and the capsule crashed into the Utah desert, breaching the sample return capsule and thus fragmenting and contaminating the solar wind collectors to varying extents. The accident made the pieces of DoS wafer an even more important target material for the precision analysis of SW. Although the depth of the SW implant in the DLC was more shallow than that of the SW in some other flown collectors, such as silicon metal, the chemical and physical properties of the DLC film made removing contamination from the surface of the DoS collectors relatively easy and, therefore, made those collectors a prime target for SW analyses.

### Amorphous diamond-like carbon films

**Figure 1:**
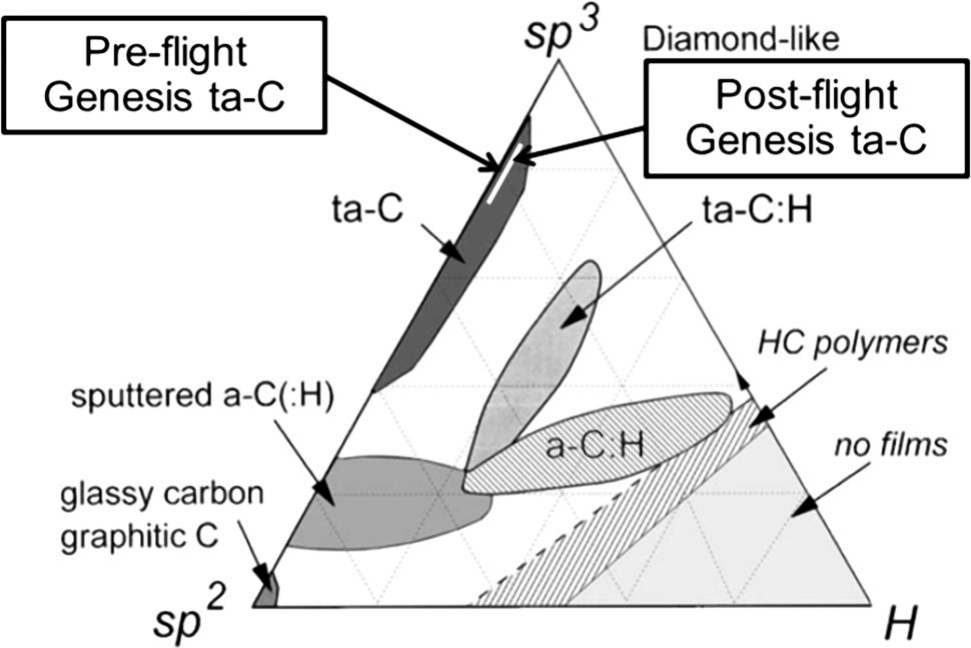
Relationship of Genesis material to other forms of amorphous carbon films as a function of bonding and H-content (after [4]). Note that even the compositions corresponding to the peak of the Genesis SW H implant (*white line*) plot well inside the tetrahedrally bonded amorphous carbon (ta-C) field. An overview of characteristics specific to each field is given in [4]

The phrase “diamond-like carbon film” represents a suite of engineered materials, consisting of “amorphous” C with varying amounts of H, N, Si, and/or other dopants used to control specific properties. This material is used for diverse purposes: from reducing friction and increasing wear resistance to vacuum field emission devices [4, 5]. A detailed review of diamond-like carbon films, manufacture, properties, and uses is given by Robertson [4]. Figure 1 shows a schematic ternary of *sp* ^2^–*sp* ^3^–H bonding (after [4]) illustrating the relationships among carbon-based films which are of most interest in engineering. As a general rule, *sp* ^3^ (fourfold, “diamond like”) bonding adds strength and wear resistance, increased thermal conductivity and lower electrical conductivity relative to *sp* ^2^ (threefold, “graphitic”) bonding; incorporating H into the carbon structure during fabrication can allow increased diffusivity, elasticity, and polymerization. H exposure in the laboratory can, however, change the amount of *sp* ^3^/*sp* ^2^ carbon bonds at the surface, with the direction of that change depending upon environmental conditions [6]. Damage to DLC under ion beams has been known to catalyze non-equilibrium changes in microstructure (either graphitization or diamond growth) by altering the local stress state of the film. Significant radiation damage of DLC in the laboratory can also be used to damage the amorphous structure directionally, to form graphene [7].

The DLC flown on the Genesis spacecraft was made by Sandia National Laboratories using procedures discussed in ([5], [8] and references therein). Preflight it was an anhydrous, tetrahedrally coordinated carbon film, with >50% *sp* ^3^ bonding, while postflight hydrogen is ~0.5 at.% in the radiation-damaged area, which is easily measurable by SIMS [3]. The approximate compositions of both the preflight and postflight Genesis films are plotted on the schematic in Fig. 1.

The Sandia DoS wafers were selected as Genesis collectors because of their composition and chemical properties [9]: carbon has low backscatter during implantation, and this DLC is retentive of volatiles but very low in H and other volatiles. Moreover, for secondary ion mass spectroscopy (SIMS) analysis, molecular ion interferences from carbon matrices are minimal. The caveat to using DoS wafers was that the amorphous film was structurally inhomogeneous. The assumption was that those variations were nanoscale and dispersed relatively uniformly throughout the film, and our (150 × 150 µm) or greater analysis area should give a consistent sample for SIMS analysis.

When analyzing SW in DoS collectors using SIMS, we made our conditions as stable as possible and used analytical reference standards made from preflight material. We sputtered 250 × 250 µm areas using the center 150 × 150 µm square to measure composition. Still, our SIMS data on the Genesis DLC proved to be difficult to reproduce, with measured compositional variations much larger than calculated using counting statistics. So, was the intrinsic inhomogeneity of the DLC film on a scale larger than 150 × 150 µm, and did the properties of the amorphous film change due to the implantation of the SW?

Here we investigate variations in the physical and electronic properties of the DLC for areas of approximately 2.3 × 10^−4^ cm^2^. We do this by doing depth profiling by SIMS analysis on both our analytical standard (commercial implants in preflight DoS which was not flown) and SW implants (the “Genesis-flown” collector). Then, assuming that variations in the standard analyses record representative “preflight” inhomogeneity, we compare the results of the Genesis-flown sample with the standard implant to see whether SW exposure caused other structural, as well as chemical, variations.

### Analysis by secondary ion mass spectroscopy

An overview of the uses of SIMS analysis in materials engineering is given in [10]. Concepts relevant to this specific work are summarized below.

For general chemical measurement by SIMS, a primary ion beam breaks down, mixes, and ejects the material at the surface of a sample. This material is ejected as molecules, atoms, and ions; the electric field of the sample then accelerates the secondary ions for analysis with a mass spectrometer. There, the signal of the ion of interest is measured relative to the signal of an ion of matrix material (^12^C^+^ in our case). A thorough disassociation of the matrix is necessary to minimize molecular interferences and obtain a reproducible, large signal-to-noise ratio (e.g., “ions of interest” to matrix ions). Even so, molecular ions (charged, semi-coherent clusters of atoms) can sometimes be generated directly from the matrix. The intensity of a molecular ion depends upon the material being analyzed and the analytical conditions, including (1) the energy at which ions from the primary beam impact the sample, (2) how the ions from the primary beam chemically interact with the sample, and (3) the composition of the sample and strength of its bonds.

Depth profiling is a type of chemical analysis by SIMS [10] in which the instrument is configured so that concentration changes are measured as a function of depth. For quantitative depth profiles, the depth of the sputtered pit must be measured (here by stylus profilometry), which allows the sputtering rate to be quantified (e.g., (pit depth)/(analysis time) for constant current). Moreover, the sputtering process must minimize the impact energy of the primary ions to minimize ion mixing in the concentration profile; yet the impact energy must be high enough to be in the dynamic SIMS range [10].

To quantify the elemental information from the mass spectrometer, it is assumed that the signals (measured in counts per second (cps)) from the sample are proportional to the signals from a characterized standard. Specifically, the relative sensitivity factor (RSF) is a proportionality constant used. It is defined as the *inverse* of the ratio of the secondary ion signal to its matrix ion signal for a known concentration. (Normalizing the secondary ion signal to a matrix ion implicitly corrects for unavoidable variation in analysis conditions, e.g., minor drift of the primary beam current). So, by multiplying the matrix-normalized secondary ion signal of an unknown by its RSF (as determined from the standard) you get the concentration of the unknown [10, 11].

When either the standards or the unknown are implant profiles (cf., [11]), the concentration is the integral of the entire concentration profile (counts per unit area) with depth, where the area is the analyzed area in cm^2^. That is, RSF is written:

$$ RSF = \left( {fluence\,of\,standard} \right)/\left( {\mathop \int \limits_{0}^{x} \left( {\frac{cps\,element\,of\,interest\,in\,standard}{cps\,matrix\,in\,standard}} \right)dx} \right) $$

where dx is the change in depth for each SIMS duty cycle (when peak jumping) and x is the depth of the analysis crater.

RSFs change with the analytical conditions, so there could be issues relying on RSF when the drift in an analytical parameter is extreme, such as large changes in beam current or oxygen pressure. RSFs can change with subtle variations in matrix properties so care must be taken that SIMS standards are truly matrix appropriate (cf., [11]).

## Experimental details

For this study, “Experimental” covers three distinct subject areas. The first topic, ion implants, details both the Genesis SW implant and the calibrated reference implant used as a SIMS standard. The second topic, SIMS conditions, discusses collection of the data. The third topic is the use of SRIM for understanding the structures in the DLC by comparing the models with SIMS results. Technical details for each topic are discussed separately in this section.

### Ion implants

Two implants into Genesis DoS were used in this study: the Genesis solar wind sample and a commercial implant into Genesis flight-spare material. Details are given below.

#### The Genesis SW sample

An overview of the Genesis Mission is given in [1], and the SW implant is detailed in [2]. However, the salient points to note about the Genesis-flown DoS fragments used here are:

1. Temperature of SW collection is not precisely known. However, engineering documents in the Mission archives estimate that the temperature for DoS was <160 °C.
2. The SW in the collector fragment (DoS #20732-2) was bulk solar wind (B/C array) passively collected for 27 months.
3. SW implant was primarily H at a dose of 2.06 × 10^16^ ion-cm^−2^ with SW He implanted at a He/H ratio of 0.0391. All other elements had dose orders of magnitude less than that of He [2].
4. SW ions were implanted at a range of energies which depended on mass, charge, and velocity. The observed peak concentration of H depth profiles in DLC is ~12.0–14.0 nm below the surface [3]; the peak He concentration was ~20.0–23.0 nm [2]. These parameters were monitored by instruments aboard the Genesis spacecraft as well as other in situ spacecraft ([2] and references therein) so that the energy spectrum of the SW implant is known (e.g., [12, 13]).

The original intent of our SIMS analysis on these solar wind-implanted samples was to measure the Mg isotope ratios in the solar wind [14]. Accordingly, for analytical quantification, a two-isotope Mg implant was made at a commercial facility.

#### The calibrated reference implant (standard)

Genesis flight-spare silicon and DoS wafers were mounted on a plate and co-implanted (as per [11]) with ^25^Mg and ^26^Mg at Kroko, Inc (Tustin, CA), each at a nominal fluence of 1 × 10^14^ ions-cm^−2^ at an energy of 75 keV. The actual ^25^Mg/^26^Mg ratio of the implant (^25^Mg/^26^Mg = 0.976 ± 0.005) was determined by ICPMS on an aliquot of the implanted silicon. The fluence of the Mg species was calibrated using a co-implant into silicon and a NIST glass with known Mg content (SRM 617 with Mg quantified independently by ICPMS [11], [15]). Using SIMS depth profiling in silicon to calibrate our sample relative to the implanted glass, the ^26^Mg fluence in our reference implant standard was determined to be 3.8 × 10^13^ ions-cm^−2^. There was also a small “accidental” ^24^Mg implant profile. This “accidental ^24^Mg implant is because naturally occurring Mg is ~90% ^24^Mg, and the vendor is not able to fully resolve the signal of ^24^Mg–H from that of ^25^Mg for implantation.

Fortuitously, in the standard, the doses of the “accidental” ^24^Mg implant and the ^26^Mg implant bracket the magnitude of the ^24^Mg dose in the SW. That is, the SW ^24^Mg is ~5× that of the ^24^Mg in the standard, but ~4× less than the ^26^Mg of the standard. Moreover, the minor isotopes ^25^Mg and ^26^Mg in the SW have doses ~2× the ^24^Mg implant in the standard. The low dose of the ^24^Mg implant in the analytical reference standard has made it useful for a number of purposes. For this study, it was primarily used for identifying terrestrial Mg contamination occurring both on the surface of the film and as features within the DlC film.

### SIMS conditions

#### General configuration

Depth profiling of the Genesis DLC films used the CAMECA IMF-6f instrument at ASU. Analytical details include a 22 nA O_2_^+^ primary beam with an impact energy of ~7.5 kV and a 250 × 250 µm raster. Sputtering rates were ≤0.03 nm sec^−1^. This SIMS configuration was selected because the depth resolution was sufficient to reject signal from terrestrial surface contamination and resolve differences in the implantation depths of different solar wind isotopes.

The DLC film in the implant standard was analyzed exactly as were the films from the Genesis DoS. Because the SIMS configuration was optimized for SW analysis, i.e., an approximately 25 keV implant of the Mg isotopes, each profile of the 75 keV implant standard took over 7 h to run. However, primary beam current was stable, and keeping all of the parameters identical ensured consistency of RSFs (assuming homogeneity of the DoS).

To mitigate the chance of sampling stray terrestrial Mg ions from the surface of the wafer fragment or the upper walls of the analysis pit when analyzing at depth in the film, both “60% DTOS” and a field aperture were used. “DTOS” stands for “Dynamic Transfer Optical System,” a CAMECA function which focuses the secondary ions from each position of a rastered beam to a point, allowing a concentrated signal to be sent to the mass spectrometer. “60% DTOS” refers to a modification of DTOS in which an electronic gate allows only secondary ions from the inner 60% of the rastered crater to be concentrated. That is, for a 250 × 250 µm raster, the analyzed area is 150 × 150 µm. Note that the electronic gate serves the traditional role of a field aperture for a rastered beam. In practice, however, neither the primary beam nor the secondary beam is focused to exactly a point, and the sputtered crater bottom is never ideally flat. So, in addition to 60% DTOS, we added a field aperture to circumscribe the focused secondary beam in order to mask any scattered secondary ions. The field aperture did not affect the nominal analyzed area.

We wanted to maximize the signal from the solar wind while minimizing the chance of collecting ions ejected from the crater surface (terrestrial contamination) or the walls of the crater. To accomplish this, we maximized the analyzed area within the 250 × 250 µm primary beam raster. If we increase this parameter too much, we will sample the crater walls (or worse, the upper edge of the crater which includes the surface and, therefore, ion-mixed surface contamination)! If we make this too small, then the Mg signal intensity decreases to values too low for precise estimates of the isotope ratio. After several tests, we set the CAMECA SIMS system to allow secondary ions from 60% of the rastered area (the inner 150 × 150 µm region) into the mass spectrometer using the dynamic transfer optical system (DTOS) set to 60%. We observed that this instrument setup did not give optimum flat-topped peaks on the isotopes of interest. We then inserted a 750-µm-diameter “field aperture” in the image plane of the secondary ion optical system. Although this decreased the total signal by a factor of ~2, it provided the optimum peak ion intensity for the Mg in the implant standard; ie., the highest signal from the implant which did not require a significant correction for detector dead time. This setup also provided the optimum mass resolution to separate ^24^Mg^+^ from the ^12^C_2_^+^ ion (see below).

#### Details of measurement

Other relevant settings and procedures were as follows. Mass spectra on a ^25^Mg–^24^Mg–H implant had previously shown that ^24^Mg–H secondary ions in DLC were insignificant [14]. This allowed us to relax the mass resolving power (MRP) to conditions where C_2_^+^ was fully separated from ^24^Mg^+^ (M/∆M ~ 1600). If the ^24^MgH ion were present, MRP of >3500 would be required which could be obtained only by closing the entrance slits to the mass spectrometer and reducing our signal by at least 2×. A CAMECA 9-hole mount held the samples. Depth profiles were run on DLC held in the center hole of the mount at least ~1.3 mm from the metal in order to avoid non-uniform extraction fields. Depths of analytical pits were measured using the ASU KLA-Tencor Alpha Step 200 profilometer. Sputtering rates reported are the depths of analytical pits divided by the total time for the analysis.

^12^C, ^12^C_2_, ^24^Mg, ^25^Mg, and ^26^Mg were collected sequentially by mass peak jumping. That is, only one species of ion was counted at a time: the signal of each ion reported for the “duty cycle” of the SIMS analysis (cf., RSF, “Analysis by secondary ion mass spectroscopy” in Background) is based upon the fraction of time that species was counted during that analytical cycle. Linear interpolation was used to insure that the signals reported represented the same depths. However, given the very slow sputtering rates (≤0.03 nm s^−1^) and the relatively short duty cycle (~13 s) used in this work, the concentration of each species was fairly constant over most of the duty cycles. Depth profiles into the 75 keV implant measured secondary ions for all five species (by peak jumping) throughout the analysis. Depth profiles into SW implants measured the ^12^C and C_2_ only at the beginning and end of each profile to increase time available for measuring Mg species.

Both the ^12^C^+^ and C_2_^+^ measurements on the Genesis collector were taken manually, in depth profile mode, for times ranging from ~18 s to 270 s, but the majority of the measurements were 25 s for both initial and final matrix ion counts. Note that ~25 s of sputtering at the beginning of a SW depth profile with a nominal sputtering rate of 0.03 nm s^−1^ consumes only ~0.08 nm of sample. Accordingly, this matrix measurement generally did not substantially affect the depth profile obtained for the Mg species.

#### Reduction of data

For calculating errors, the raw signal in cps collected for each ion does not account for the instantaneous count rate or dead time of the electron multipliers. The correction for instantaneous count rate was an empirical factor of 2.8 for these analytical conditions; dead time corrections were small. However, although statistical error calculations required total counts, the data reported here are as measured (i.e., raw, unprocessed intensities) unless otherwise specified.

For identification of Mg-rich features in the DLC, as well as surface contamination, all three isotopes of Mg were measured. To be validated as an anomaly in the collector and not a SIMS artifact, the feature had to be present in all three isotopes, and the ratio of those isotopes had to be within error of the known terrestrial value.

For plotting depth profiles, the depth scale is calculated by multiplying the time of acquisition by the sputtering rate (S). Here, S is assumed linear and is determined by dividing the measured depth of the analysis pit by the total time of the analysis. SIMS data in most figures are “normalized”; that is, the counts per second (cps) of the Mg species (^24^Mg, ^25^Mg or ^26^Mg) or molecular ^12^C_2_^+^ are divided by the ^12^C^+^ matrix ion cps. Normalization eliminates any change in the secondary ion signal caused by minor drift in the primary ion current.

For calculating derived parameters, empirical factors include the primary current-normalized sputtering rate, shape factors for the depth profiles, and relative sensitivity factor. These parameters include implicit assumptions in their derivation, such as constant beam current or sputtering rate. For example, sputtering rate per nanoampere (S/nA) was calculated for each depth profile by dividing the calculated S by the average primary beam current. Shape factors (for depth profiles of Mg species) were the depth of the peak concentration (X_peak_), the depth in the sample (on the deep side of the peak of the profile) at which the concentration falls to half of the peak concentration (X_half_). These shape factors were calculated as follows. The peak depth was calculated by fitting the implant peak with a sixth-order polynomial and then taking the derivative. The half-height calculation used the peak concentration as defined by the polynomial and the depth versus normalized SIMS Mg data (linearly extrapolating between steps due to the SIMS duty cycle). The relative sensitivity factor was calculated as in “Analysis by secondary ion mass spectroscopy” in Background.

### SRIM modeling of amorphous diamond-like carbon

SRIM is a freeware program [16, 17] generally used to design experiments and to engineer materials exposed to keV-MeV radiation. That is, SRIM is used to predict the concentration profile of an ion implant into a designated material. Variable parameters include the composition of layers, thickness of layers, density, and bonding. For modeling when the specifics of the bond energies are not known well, SRIM allows variation of an empirical constant for modeling overall stopping power. Here we used SRIM to estimate properties such as the local bulk density and bonding of our DoS films from SIMS-generated implant depth profiles.

The impact energy of impinging ions is also a variable in SRIM. For this study, that feature was used to model what we would see if the impact energy of the primary beam was reduced by local buildup of charge in a highly insulating area of the sample. The SRIM results were then compared to observed depth profiles.

## Results

Results include SIMS data and SRIM model calculations, as well as SEM and interferometry observations. Also presented are empirical parameters calculated for individual analyses, as described in “Reduction of data” in Experimental Details. *Each of these parameters should be constant within counting statistics for single implant and constant analysis conditions in a homogeneous material.* So, for this study, irreproducibility ideally reflects inhomogeneity.

### Standards

#### Mg implant results

**Figure 2:**
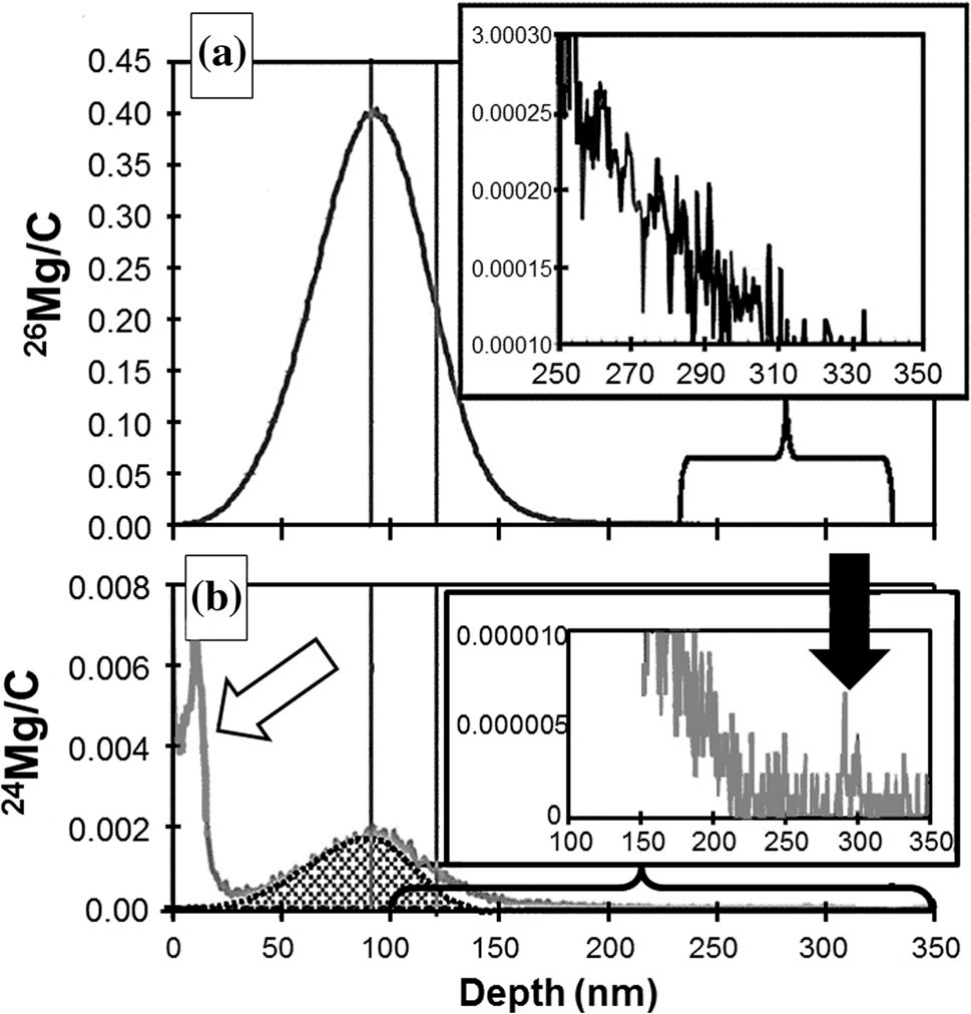
**a, b** Mg depth profiles from standard analysis Profile 5. Mg cps normalized to ^12^C^+^ cps (for each SIMS duty cycle) is plotted versus depth below the collection surface. ^12^C^+^ was measured throughout the profile. Two *vertical lines* (in both **a**, **b**) indicate the peak and ½-peak of the ^26^Mg/^12^C profile. Errors for X_peak_ and X_half_ (calculated by varying integration parameters) are ~0.3 and ~0.1 nm. **a** ^26^Mg implant profile. Note: the ^25^Mg implant profile (not plotted) would be nearly indistinguishable, **b** “accidentally implanted” ^24^Mg profile. *Shaded* region: a SRIM model implant into a “#906 nuclear grade graphite” layer with a density of 3.0 gm cm^−3^ (as per [5]). Non-uniformities indicated by *arrows* (open = embedded Mg-bearing particulate; filled = Mg-rich annealing surface)

Figure 2a and b plots ^12^C^+^-normalized ^26^Mg and ^24^Mg, respectively, from a single analysis of the commercial reference implant. The purpose of the insets are to magnify variations in the low-intensity signal from the tail of the implant, the depth highlighted by the inset is designated by the respective bracket.

Two types of inhomogeneous Mg background are identified in Fig. 2b, the (low-intensity) ^24^Mg profile. The open arrow points to a near-surface anomaly, probably a small particulate entrained in the DLC (it wasn’t observed in the ion image before sputtering, only during analysis). The filled arrow points to a small fluctuation in Mg identified as terrestrial Mg of a type occasionally seen in blank material or when the implant’s signal is negligible. For reference, the terrestrial contamination signal indicated by the filled arrow is small relative to the SW Mg signal and so should have little if any effect on the measurement of SW Mg. In contrast, although negligible with respect to the high ^25^Mg and ^26^Mg concentrations of the analysis standard, contamination from embedded particulates has been problematic for SW Mg measurement [18].

Figure 2 also plots the shape factors calculated for the SIMS depth profile (standard Profile 5) and shows how they can be used to assess the fit of SRIM models. The two vertical bars (Fig. 2a, b) illustrate the X_peak_ and X_half_ positions of the ^26^Mg data (cf., "Reduction of data" in SIMS Conditions). The shaded region in Fig. 2b is a SRIM simulation for a ^24^Mg 75 keV implant into “#906 Nuclear Grade Graphite,” one of the SRIM compound options, having a density of 3.0 g/cm^3^ as reported by [5]. Note that the X_peak_ calculated for the ^26^Mg looks reasonable for this SRIM model, but the X_half_ does not.

**Figure 3:**
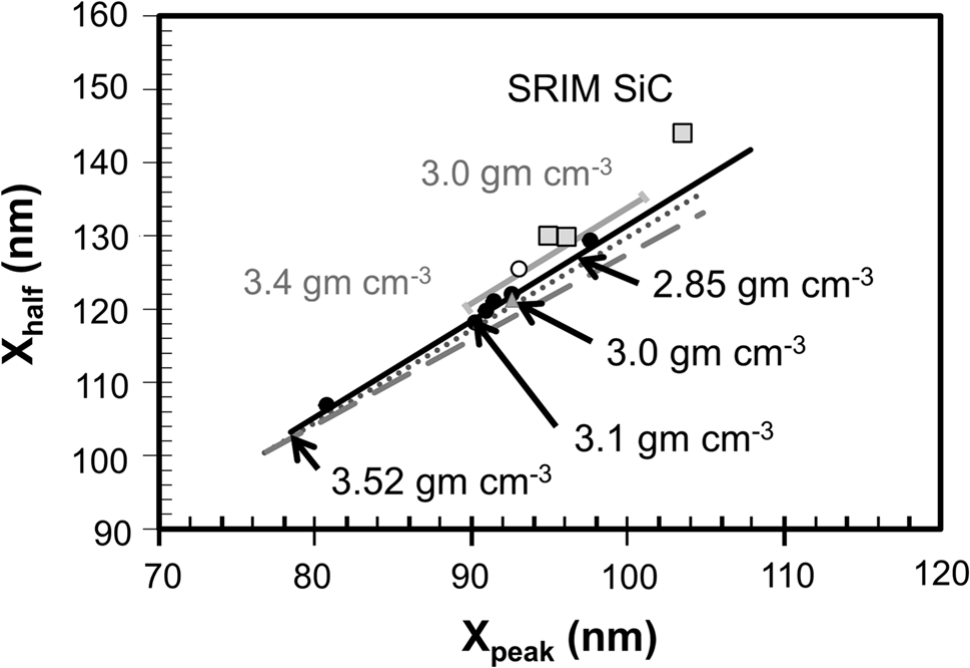
Comparison of shape factors for SIMS profiles (*filled circles*) in the standard with those of various SRIM models which separately vary density, bonding and composition. *Lines* are best fits to SRIM calculations varying density pure carbon matrices; each *line* represents a matrix with a specific bond parameter (*solid line* = SRIM catalog #906 Nuclear Grade Graphite matrix = carbon with an empirical compound correction of ~0.866; *dotted line* = carbon using the settings which SRIM uses for carbon in crystalline SiC; *dashed line* = default settings which SRIM uses for carbon, *gray line* = carbon with a compound correction of 0.6). *Black numbers* with *arrows* point to the approximate density for four positions on the *black line*. *Gray numbers* represent the densities used for the endpoints of the *gray line* (note the shift from densities of #906 nuclear grade graphite). *Gray-filled markers* are SRIM models in which some percentage of Si (as SiC) was added to the carbon layer: *triangle* (partly hidden by 3.0 gm cm^−3^ *arrow*) = model results for ^26^Mg at 75 keV into carbon using the default carbon setting +7% Si; pure SiC is marked. Note: a given (X_peak_, X_half_) can be modeled in several ways in the absence of independent composition, density and bonding information. *Open circle* = variable-sputtering model of Profile 4 discussed in “Standard Profile 4” in the Discussion and the Online Reference (SOM)

Figure 3 compares the shape factors (cf., “Reduction of data” in Experimental Details) for all six standard implants with the results of SRIM models. The first thing to note is that the measured data do not overlap; therefore, the DLC is inhomogeneous on the scale of compositional data collected from a 150 µm × 150 µm area. Each line in Fig. 3 represents a best fit to a set of SRIM ^26^Mg 75 keV implant calculations run for different densities of a homogeneous, single-layer material. Each line assumes a matrix having 100% carbon, but with different C–C bonding. For each material and each density, multiple models were calculated, and then lines were fit to the results. The linear fit was necessary because the 75 keV implant is deep enough that the automatic binning of data in the SRIM output [16] added error to individual models. Fitting a line to data mitigated those systematic errors. The carbon matrices (differing only in bonding) were: (1) SRIMs “#906 Nuclear Grade Graphite,” (2) carbon using the SRIM default settings, (3) carbon using the lattice energy settings for carbon which SRIM uses (only) for the carbon in crystalline SiC, and (4) carbon with a hypothetical SRIM compound correction of 0.6. Again, since all three materials are carbon matrices, differences in the slopes of the lines reflect differences in bonding. However, we note that another SRIM model (triangle, Fig. 3) suggests that the “#906 Nuclear Grade Graphite” SRIM results are also consistent with carbon containing ~7% Si as silicon carbide.

It is important to recognize that the consistency in the results between the #906 Nuclear Grade Graphite and the 7% silicon-containing carbon does not necessarily mean that the #906 Nuclear Grade Graphite contains silicon. While it may indeed have some silicon (silicon is a ubiquitous minor component in commercial graphite, e.g., [19–21]), you might also expect “Nuclear Grade” to be more pure than 93% carbon. In the compilation of targets, SRIM catalogs #906 Nuclear Grade Graphite is listed as a pure carbon compound, having bonding energies determined empirically through the SRIM 2008 feature “compound correction.” This feature adjusts the stopping power for observed non-ideality, but does not define what makes the matrix non-ideal. So, what this consistency does indicate is that, for our DLC data (solid circles in Fig. 3), SRIM models may be non-unique. The density, composition, and bond energy required for displacement and damage of the matrix may be varied independently in SRIM, and so the average bonding (and, therefore, average stopping power) may be changed by undocumented features of the material.

In Fig. 3, densities are marked on the “#906 Nuclear Grade Graphite” line. Because a best fit was required (and because SRIM results are non-unique, within tight constraints) applying these densities to our DLC is an approximation. Comparison of our plotted data with that particular model suggests that columns of our DLC range in nominal density from ~3.4 to ~2.85. (Note: the X_peak_ and X_half_ for each standard analysis are given in Table 1, which also includes other measured and derived parameters such as primary current, ion yields, and sputtering rate.) Other data, mostly SRIM models containing differing amounts of Si as SiC, are included in Fig. 3 for reference.

**Table 1:** Summary of relevant parameters measured and calculated from reference implant standard

Implant* analysis	^12^C^+^ (cps)	Error (1σ)	^12^C_2_^+^ (cps)	Error (1σ)	Primary (nA)	|Initial–final| (nA)	^12^C_2_^+^/^12^C^+^ (average)	Error (1σ)	S/nA (nm/sec‐ nA)	Error (1σ)	X_peak_ (nm)	X_half_ (nm)	^26^Mg RSF (ions cm^−2^)
2	61843	0.4%	8313	1.1%	23.825	7%	0.1344	0.2%	0.00127	0.04%	90.2	118.2	2.91 × 10^19^
3	79484	0.4%	10915	1.0%	21.67	0%	0.1373	0.1%	0.00123	0.05%	80.7	106.9	3.17 × 10^19^
4	22143	0.7%	3401	1.7%	17.36	8%	0.1536	0.3%	0.00122	0.06%	97.6	129.4	2.78 × 10^19^
4b	nd	–	nd	–	(17.69)	(26%)	nd	–	0.00122	–	93.0	125.5	2.72 × 10^19^
5(a)	50864	0.4%	6750	1.2%	22.855	12%	0.1327	0.2%	0.00104	0.04%	92.6	122.1	3.14 × 10^19^
6	62807	0.4%	8660	1.1%	23.155	12%	0.1379	0.2%	0.00105	0.04%	91.4	121.1	2.90 × 10^19^
7	56476	0.4%	6701	1.2%	23.47	13%	0.1187	0.2%	0.00098	0.04%	90.9	119.8	3.58 × 10^19^

* Matrix cps corrected for instantaneous count rate and dead time, and ^26^Mg RSF has non‐systematic errors ≪0.01%; (a)^12^C ^+^,^12^C_2_^+^ taken at the end of the analysis only

*nd* not determined. See Fig. 7 for details of raw count rates

**Figure 4:**
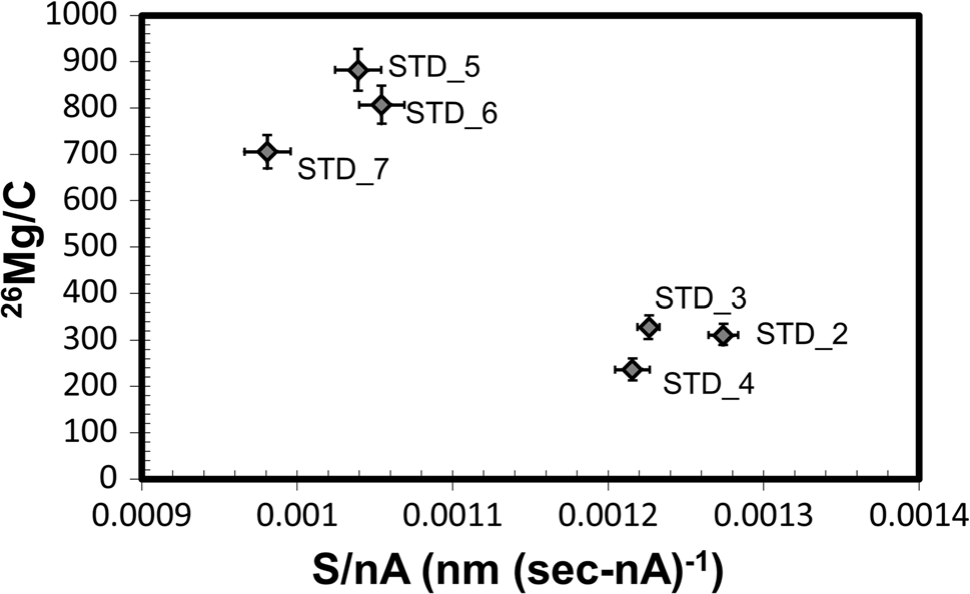
Normalized total (integrated) ^26^Mg intensity versus the sputtering rate normalized to the average beam current (S/nA) for each implant analyses. Labels designate standard analysis profile number. *Error bars* for ^26^Mg/C are 1σ of counting statistics; error on S/nA is primarily due to current drift. For a uniform material, these data would plot to a single point within the errors, instead the data groups loosely as two clusters, and there may be significant variation within the clusters

Figure 4 plots the integrated, matrix-normalized ^26^Mg counts (^26^Mg/C) versus current-normalized sputtering rate (S/nA) for the analyses of the implant standard. Note that ^26^Mg/C is an ion yield for a 150 × 150 µm area, while S/nA gives a normalized rate for removal of material from a 250 × 250 µm area. For a uniform material, these parameters should be constants, within error. Instead, these data fall into two loose clusters: one having a low sputtering rate per nA with a high secondary ion yield and one having a relatively high sputtering rate per nA with a low secondary ion yield. Moreover, at the two-sigma level, Profile 7 is different than Profiles 5 and 6, suggesting possible variations within the clusters as well.

To minimize the complexity of Fig. 3, the profiles were not labeled. However, the clusters in Fig. 4 can be compared with the dots in Fig. 3 using the X_peak_ and X_half_ values listed in Table 1. In Fig. 3, Profiles 5, 6, and 7 plot near the 3.0 gm cm^-3^ marker as well as near the gray triangle(SRIM model of 7% Si in carbon: default C bonding parameters used). Profiles 2 and 3 plot at higher densities on the solid line; Profile 4 plots at ~2.85 gm cm^-3^. Despite their similarity with regard to ^26^Mg/C and S/nA in Figs. 3 and 4, Profiles 2, 3, and 4 show very different “stopping powers” (e.g., density, composition, and/or bonding differences); conversely, Profiles 5, 6, and 7 plot similarly in both figures.

#### Matrix results

Direct information from the DLC matrix comes from SIMS depth profiles of ^12^C^+^ and ^12^C_2_^+^ ions, as well as through inspection of the SIMS pits via scanning electron microscopy and optical interferometry. It should be noted that (1) profiles composing the high sputter rate, low ion yield cluster of Fig. 4 came from a relatively small area (about 2 mm in diameter), while the profiles composing the low sputter rate, high ion yield cluster were scattered across about a centimeter of DLC. It should also be noted that, although the plan was to take ^12^C^+^ and ^12^C_2_^+^ data throughout all of the analyses on the standard, when we conducted Profile 5, we mistakenly collected these ion intensities for only the first 18 s of the analysis.

##### Depth profiles

**Figure 5:**
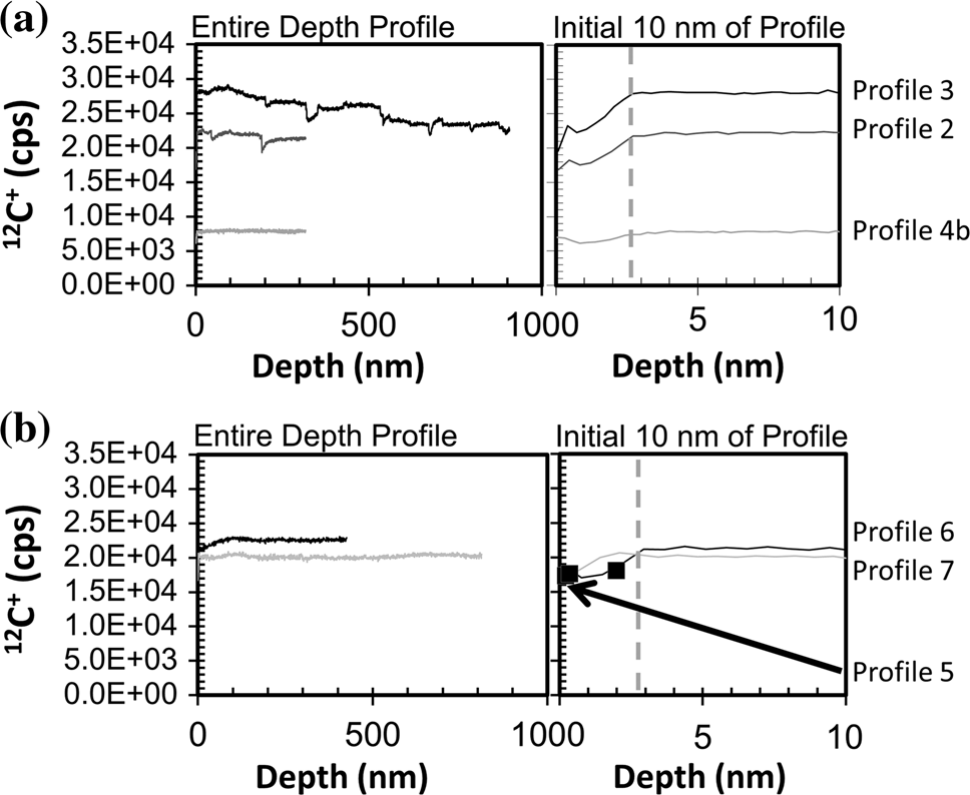
**a, b** Raw ^12^C^+^ intensity versus depth for each profile plotted in Fig. 4 where the LHS gives the full profile but the RHS is first 10.0 nm to enhance zone of non-steady-state sputtering (observed to ~2.8 nm, marked by *dashed vertical line*). **a** high S/nA, **b** low S/nA. For Profile 5, ^12^C^+^ was only measured at the beginning of the analysis. Profile 4 is plotted versus depths calculated using a differential sputtering model; a constant sputtering model for Profile 4 extends the zone of non-equilibrium sputtering to ~9.5 nm, but the average ^12^C^+^ counts remain unchanged (detailed later in “Standard profile 4” in the Discussion and Fig. 11)

**Figure 6:**
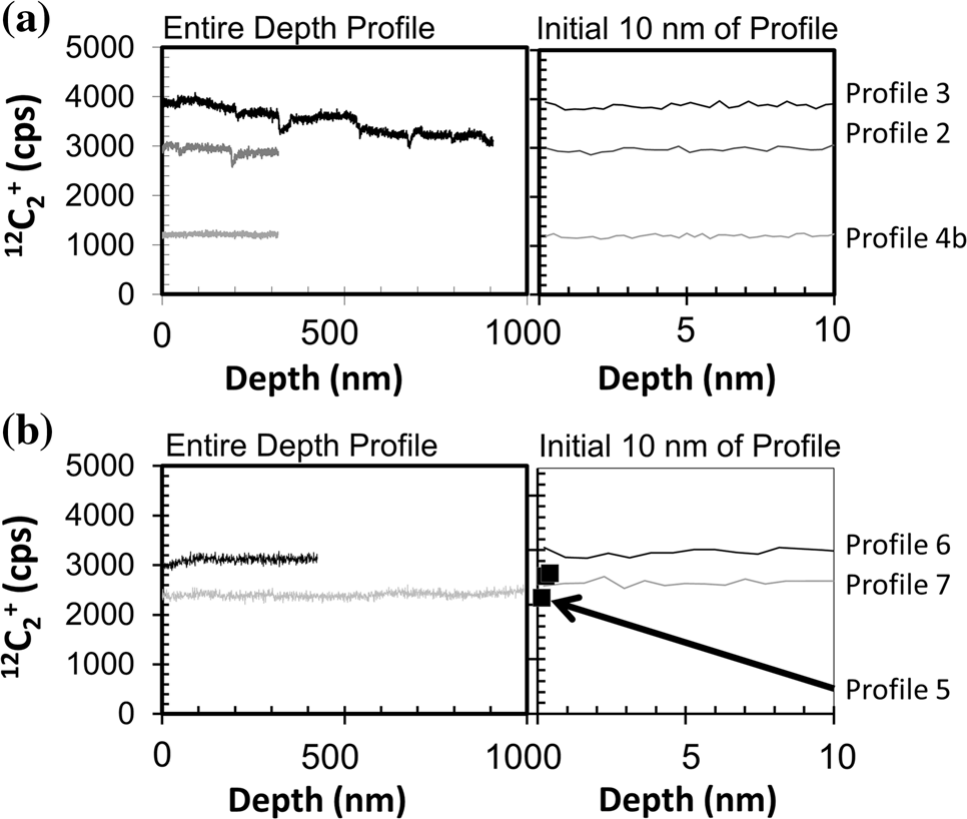
**a, b** Raw ^12^C_2_^+^ intensity versus depth for each profile plotted in Fig. 4 where the LHS gives the full profile but the RHS is first 10.0 nm. No non-equilibrium sputtering zone can be discerned; **a** high S/nA cluster, **b** low S/nA cluster. For Profile 5, ^12^C_2_^+^ was only measured at the beginning of the analysis. Depth for Profile 4 uses the differential sputtering model which affects the transient sputtering region, but average counts remain unchanged (“Standard profile 4” in the discussion, Fig 11)

In Figs. 5a, b and 6a, b, (^12^C^+^ vs. depth) and (^12^C_2_^+^ vs. depth), respectively, we compare individual depth profiles of matrix ions from the two clusters observed in Fig. 4. Figures 5 and 6 are each divided into: (1) analyses from the high sputter rate cluster and (2) analyses from the low sputter rate cluster. Moreover, the left-hand side of each figure gives the full depth profile, while the right-hand side highlights the first 10.0 nm. Within that first 10.0 nm the ion yields can change in our DLC as the multiple processes which control sputtering balance to give a steady-state ion yield (cf., [22]).

In both Figs. 5 and 6, the ion intensity is raw data (neither corrected nor normalized). Accordingly, these depth profiles are subject to minor drifts in beam current or other SIMS artifacts. For example, drifting beam current could be a reason for the general downward drift of Profile 3. In Fig. 5a, the ^12^C^+^ cps drops precipitously at somewhat regular intervals in both Profiles 2 and 3—a feature which would not be observed if the ^12^C^+^ intensity was normalized to that of ^12^C_2_^+^. These features are at somewhat regular intervals (90.0–120.0 nm), and the clear drop at 200.0 nm occurs in both Profiles 2 and 3. The same is true for the features in the ^12^C_2_^+^ intensity plots in Fig. 6a. Accordingly, whatever caused the “glitches” in the matrix species were likely a recurring feature of this portion of the sample. The other analysis in the cluster, Profile 4, does not show the precipitous drops in either ^12^C^+^ or ^12^C_2_^+^ intensity; however, it has a uniquely low matrix ion intensity throughout, being less than a third of the ion yield of Profile 3 and significantly lower than any of the profiles in Figs. 5b and 6b.

The profiles in Figs. 5b and 6b have a significantly narrower range of ion yield for both ^12^C^+^ and ^12^C_2_^+^. Moreover, the matrix intensities are stable; there are no precipitous changes in intensity—what you would expect for matrix depth profiles in a homogeneous material. However, for the zone of transient (non-equilibrium) ^12^C^+^ sputtering (RHS of Fig. 5b) the intensity of Profile 7 appears to rise above the final equilibrium value before dropping back down, while Profile 6 and Fig. 5a profiles simply rise to the equilibrium value. This deviation is >1σ of counting statistics, and there is no sign of a similar rise in the ^12^C_2_^+^ ions in the corresponding SIMS duty cycles, so it may be a sign that the matrix sampled by Profile 7 has a different concentration of a minor element (e.g., Si) or another feature affecting the approach to steady-state ion yield [22].

##### Imaging analysis pits for physical and chemical heterogeneity

To try to independently verify variations in the DLC structure inferred from the SIMS data, we examined roughness in the SIMS pits using an optical interferometer and then looked at pits by SEM in backscattered electron imaging (BSE) and secondary electron (SEI) imaging modes.

**Figure 7:**
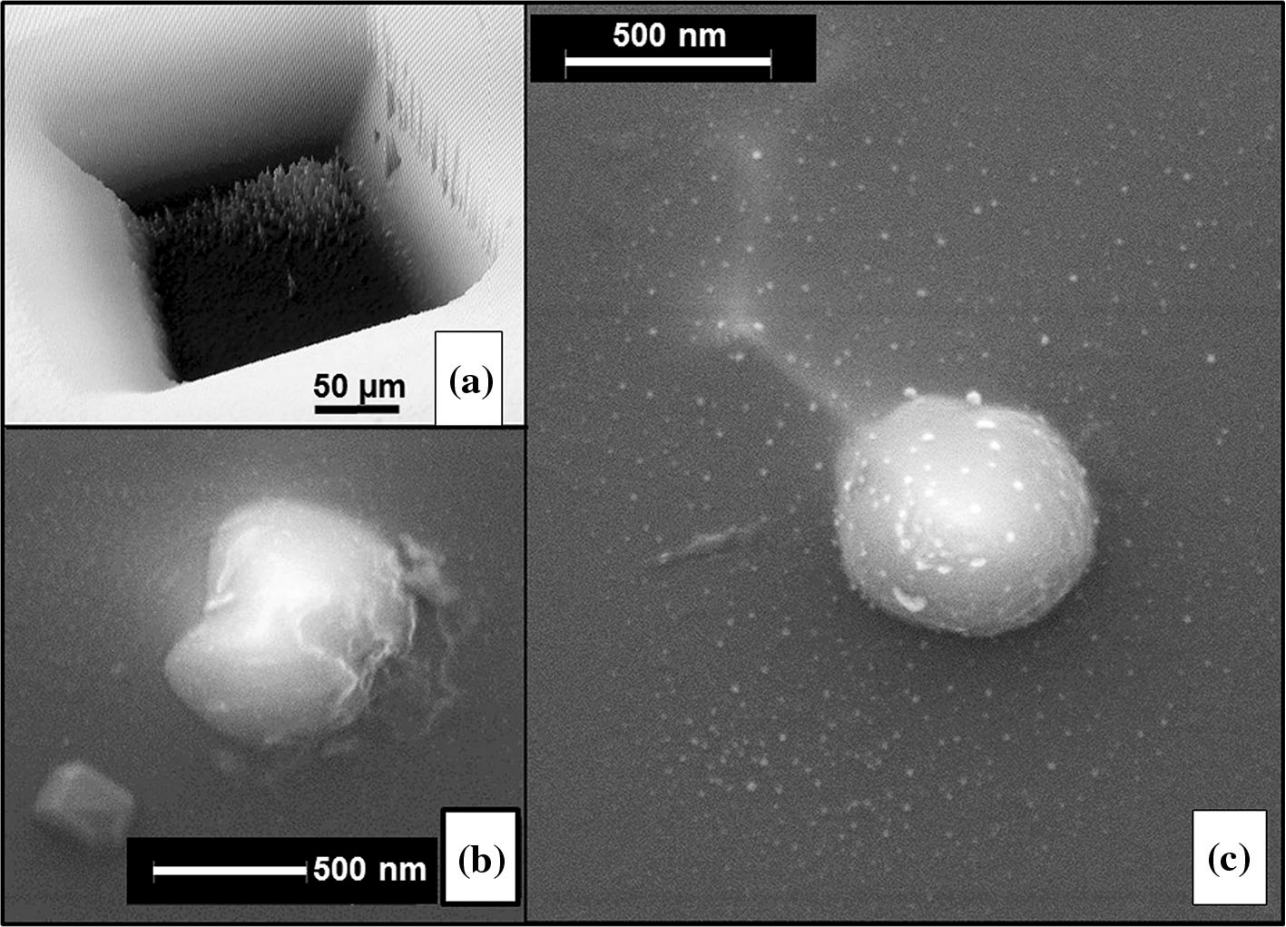
**a, b, c,** Features in floors of two SIMS analysis pits (cf., Figs. 5a, 6a). **a** Optical interferometry shows roughness but only in oblique imaging, suggesting the roughness is an artifact from multiple small areas of very different refractive index (Profile 2). **b** Crystallites imaged in SEI in Profile 3. Si and carbon were observed in the EDS spectrum, but ubiquitous Si from the substrate does not allow differentiation between diamond an β-SiC crystals. **c** A field of diamonds as seen in BEI (no Si in EDS spectrum) with large central crystal. *Bright line*(s) emanating from near large crystal are dense carbon (likely areas of diamond formation which can relieve internal stresses incurred during the fabrication of the film [24, 25]. Note the roughly hexagonal geometry reminiscent of “mud cracks”). This texture (high refractive index crystallites in a matrix) is consistent with the “roughness” seen in **a**

Optical interferometers use the polarization and reflection of light to document features on a surface. On a uniform material, optical interferometry gives an excellent delineation of surface texture. In fact, the floors of all six SIMS pits looked fairly featureless when imaged directly in the interferometer. The geometry of the few features observed on the floors of SIMS pits (lumps and depressions) suggested that these features were primarily inherited from preexisting surface roughness. However, the crater floor of Profile 2 (Fig. 7a) showed features which looked like cones often (associated with sputtering [23]), but only when observed at an angle. Moreover, an equivalent “roughening” was not observed using the SEM. We suggest that the apparent surface texture in the optical interferometer image of Fig. 7a is due to the presence of very small grains having a significantly different refractive index from the surrounding matrix. In fact, Fig. 7b and c show the images of small crystals found later when using the SEM to look for microstructures in the pits from Profiles 2 and 3.

Figure 7c shows an SEM photograph from a portion of the floor of Profile 3 which reveals a scenario especially consistent with that inferred from Fig. 7a. Figure 7c shows a backscattered electron image showing a single, large diamond crystal: close inspection shows remnant facets consistent with cubic structure (c.f. SOM); no Si was observed in the EDS spectrum, despite the proximity of the material to the DLC/Si interface. Note that the large diamond is surrounded by a field including multiple small diamonds (again, no Si in EDS analyses). The small diamonds (light or white areas) appear to add an insignificant amount of roughness, but have a very high refractive index. Also note that the light, linear regions stemming from the crystal form a set of nearly hexagonal shapes. These linear features are dense areas, probably composed of either diamond or amorphous but highly dense *sp* ^3^–bonds.

Figure 7b shows a secondary electron image of crystals elsewhere on the floor Profile 3. EDS detected Si, most likely from the substrate, but perhaps also locally. Accordingly, the compositional data and morphology (for both the beautiful octahedron and the “blob,” which charges under the electron beam) cannot distinguish whether the crystals are diamond or β-SiC. The “blob” appears to be partially sputtered by the SIMS primary beam and is about the same size as the crystal in Fig. 7c. The extensive charging suggests that it may also be diamond; however, its non-equant shape (narrow in the middle) suggests that it may be two twinned, relatively equant smaller crystals (about 1/4 µm in diameter), perhaps still partially covered by minor amounts of amorphous material from the DLC matrix.

### Solar wind results

In the SW, ^24^Mg is the major isotope in natural systems, so results are given for ^24^Mg rather than ^26^Mg (used for the implant). In addition, for SW analyses the matrix species were only measured at the beginning and the end of the analysis. This difference in analytical method for measuring matrix ions was adopted because the SW Mg signal is a fraction of that from the ^26^Mg implant, and removing matrix ion analyses increased the fraction of the SIMS duty cycle spent measuring Mg. Unless otherwise stated, the matrix ion value used in plots is ^12^C^+^_final_ or ^12^C_2_^+^_final_, the average ion intensities taken at the end of the Mg analysis. That is because ^12^C^+^_inital_ or ^12^C_2_^+^_inital_, the average of the data taken at the beginning of the analysis, are averaged signals collected prior to the crater floor reaching steady-state conditions (cf., [22] and Figs. 5, 6). Table 2 gives pertinent measured and derived parameters for SW depth profiles, such as primary current, ion yields, sputtering rate, and shape factors (X_peak_ and X_half_).

**Table 2:** Summary of relevant measured parameters of solar wind profiles

Profile	Primary nA*	^12^C_2_/^12^C^+^_initial_ **	^12^C_2_/^12^C^+^_final_ **	Ave sputter rate (nm sec^−1^)	X_peak_ (nm)	X_half_ (nm)	^24^Mg RSF (ions cm^−2^)
SW_2	20.8 (5%)	–	0.135 (1%)	0.0236	25.0	53.7	2.67 × 10^19^
SW_3	19.8 (4%)	0.202 (5%)	0.1364 (0.2%)	0.0218	20.5	47.4	2.82 × 10^19^
SW_5	21.8 (7%)	0.162 (0.3%)	0.1369 (0.4%)	0.0248	22.8	50.9	2.67 × 10^19^
SW_6	22.1 (9%)	0.186 (5%)	0.1359 (0.4%)	0.0237	23.5	50.3	2.64 × 10^19^
SW_7	20.6 (2%)	0.139 (3%)	0.125 (8%)	0.0252	19.7	50.8	3.89 × 10^19^
SW_8	21.6 (8%)	0.156 (4%)	0.126 (1%)	0.0243	24.1	56.2	2.89 × 10^19^
SW_9	21.9 (9%)	0.221 (5%)	0.140 (1%)	0.0238	24.8	51.8	2.32 × 10^19^
SW_10	21.8 (6%)	0.211 (4%)	0.146 (1%)	0.0243	24.9	72.9	2.49 × 10^19^
SW_11	23.2 (10%)	0.182 (4%)	0.124 (1%)	0.0254	23.7	49.7	2.99 × 10^19^
SW_12	23.0 (5%)	0.156 (3%)	0.122 (1%)	0.0262	13.4	33.5	2.22 × 10^19^

* Drift = (|initial−final|/average in %)

** Number in parenthesis is error (1σ)

#### SW Mg depth profiles

**Figure 8:**
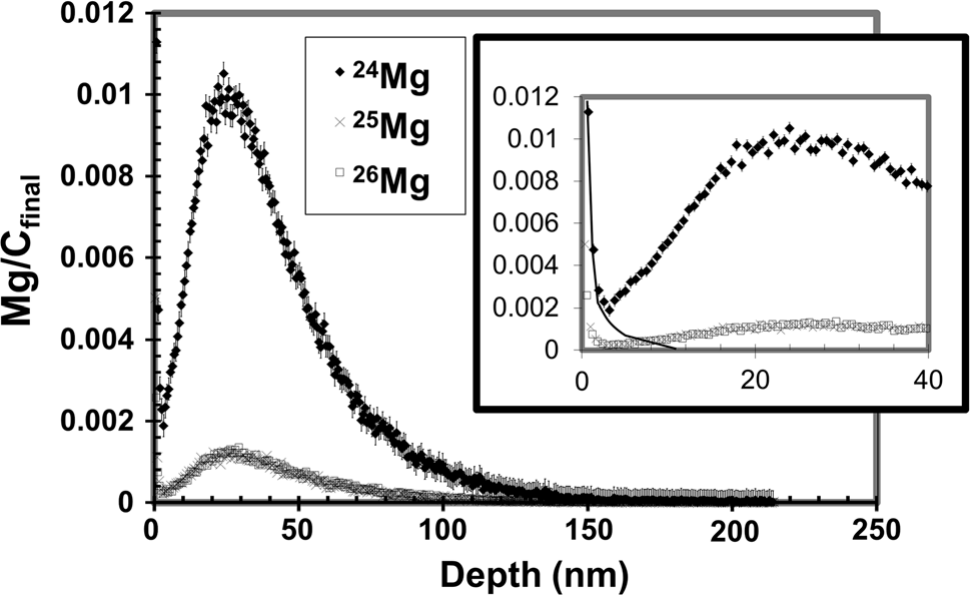
SW ^24^Mg, ^25^Mg, and ^26^Mg (cps) each normalized to ^12^C_final_^+^ (cps) and plotted versus depth. Matrix species (^12^C^+^; ^12^C_2_^+^) were only measured at the beginning and end of the SW depth profiles to increase time for counting the SW. *Inset* initial 40 nm of the profile with line to estimate ^24^Mg^+^ contributed by ion mixing of terrestrial surface contamination and drops below SW level after ~2.0 nm. Terrestrial contributions of ^25^Mg^+^ and ^26^Mg^+^ are ~ 10% of the ^24^Mg^+^. For comparison: non-equilibrium ^12^C^+^ sputtering lasts to ~2.7 nm (cf., Fig 5a, b). Mg-rich surface contamination (e.g., particulates) is obvious during analyses due to low SW Mg concentrations, cf., Fig. 2b and [18]

**Figure 9:**
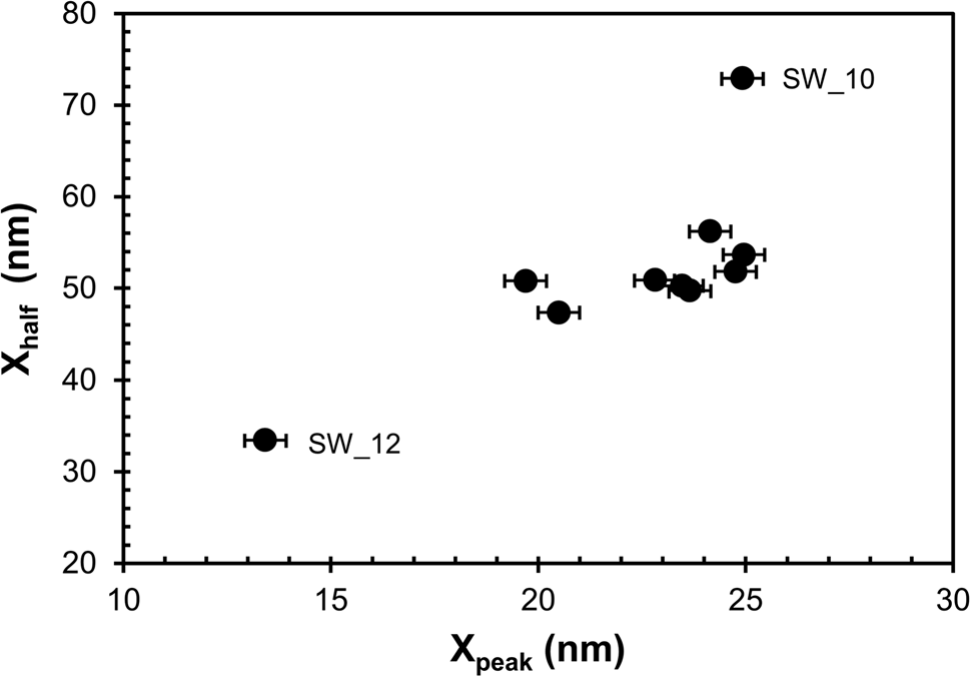
Shape parameters for depth profiles of SW ^24^Mg^+^. In a homogeneous material, these should plot to a single point. Error for shape parameter calculations is ~0.5 nm. SW Profile 12 was contaminated by a Mg-rich, near-surface particle [18], and Profile 10 may be similar to the *open circle* in Fig. 3

Figure 8 shows plots of the three Mg isotopes of SW measured in the Genesis fragment. The intensities of the Mg species (^24^Mg, ^25^Mg or ^26^Mg) were normalized to the ^12^C^+^ matrix ion, and the depth scale was calculated assuming a constant sputtering rate (i.e., (depth of pit)/(analysis time)). Note the general differences in shape and depth scale between the plot in this figure and the plot in Fig. 2a, b. (The Genesis SW Mg implant spans a range of energies having a mode of about 1 keV per atomic mass unit (i.e., ~24 keV for ^24^Mg). The standard implant in Fig. 2a, b is a mono-energetic (75 keV) Mg implant). The inset expands the initial portion of the profiles, explicitly showing that the depth to which terrestrial surface contamination is mixed into the sample by the SIMS primary beam is small. Figure 9 plots the shape factors for the SW depth profiles, which should plot to a single point (within counting statistics) for a homogeneous material. The fact that the trend is (mostly) linear is nominally consistent with trends in the standard (Fig. 3). SW Profile 12 is labeled because an embedded particle was noted near the surface [18]. SW Profile 10 is labeled because the X_half_ is much deeper than might be expected from the trend of the rest of the points and has a high ^12^C_2_^+^/^12^C^+^ (Table 2).

#### SW matrix results

**Figure 10:**
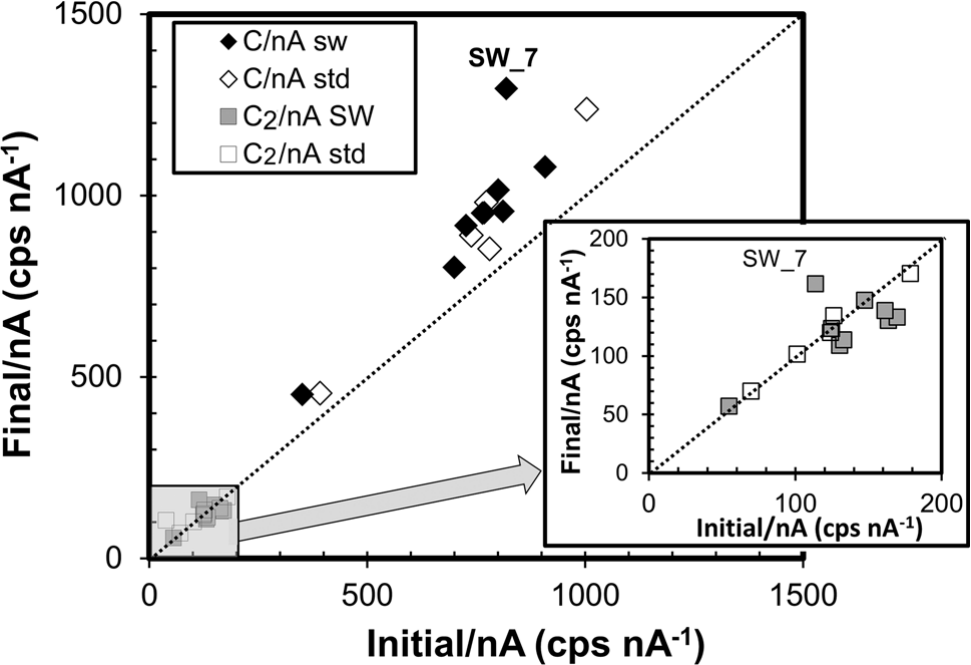
Comparison of initial versus final current-normalized matrix ion intensity (raw counts) for both SW and standard depth profiles. Markers are defined in the legend, 1σ error ≤ marker size, line designates 1:1. Matrix ions results for SW Profile 7 (*marked*) are anomalous (cf., Table 3). Note that the final ^12^C^+^ intensity is greater than the initial ^12^C^+^ intensity for both standard and collector; conversely, for ^12^C_2_^+^, the standard data fall within error of the 1:1 line, but more than half of the SW data have a relatively high initial ^12^C_2_^+^

Figure 10 gives current-normalized ^12^C^+^ and ^12^C_2_^+^ intensities from the flown collector: initial values (first 30 s) are plotted versus final values. Matrix data for the standard are included for comparison. To allow for the shorter depth of SW depth profiles, the “final” standard value was calculated for a depth of 230.0 nm, although differences between the 230.0 nm and the average value are within statistical error. For comparable matrices, the SW and standard data analyses should be statistically equivalent.

With the exception of SW Point #7, ^12^C^+^/nA for both the Genesis-flown sample and the standard fall on a linear trend above the 1:1 line. That is, there are more counts per nA deeper in the DLC than near the surface. This result might be expected because the ion intensities were collected in the non-steady-state (transient) sputtering regime shortly after the beginning of the analysis as illustrated for the standard in Fig. 5. In contrast, the ^12^C_2_^+^/nA points from the standard lie close to the 1:1 line, as expected from Fig. 6. However, only three of the ^12^C_2_^+^/nA data from the SW collector lie on the 1:1 line, while five are at least 1σ below the 1:1 line, suggesting that the relative ^12^C_2_^+^ increases near the surface. Again, SW Profile 7 has an anomalous matrix trend, as (1) both matrix species increase with depth and (2) the change in ^12^C_2_^+^/^12^C^+^ is anomalously large (Table 3).

**Table 3:** Comparing initial (first 30 s) and final matrix parameters

Profile	^12^C^+^_init_/nA	^12^C^+^_final_/nA	^12^C_2_^+^_init_/nA	^12^C_2_^+^_final_/nA	(^12^C_2_^+^/^12^C^+^)/nA change (i – f)	% change (Δ/f)
SW_2	–	1082	–	146	–	–
SW_3	811	957	164	131	0.0245	18%
SW_5	907	1079	147	148	0.0258	19%
SW_6	699	802	130	109	0.0201	15%
SW_7	819	1295	114	162	0.0727	58%
SW_8	351	452	55	57	0.0366	29%
SW_9	769	953	170	133	0.0336	24%
SW_10	764	952	161	139	0.0358	25%
SW_11	727	917	133	114	0.0326	26%
SW_12	800	1016	125	124	0.0328	27%
std_2	739	890	124	120	0.0326	24%
std_3	1003	1237	178	171	0.0401	29%
std_4	392	455	70	70	0.0237	15%
std_5	283	–	38	–	–	–
std_6	773	982	126	134	0.0262	19%
std_7	781	853	101	102	0.0104	9%

Data not corrected for instantaneous count rate/dead time

–, not determined

## Discussion

The parameters calculated from the SIMS data (above and in the tables) were chosen such that each should be a single value for a replicate measurement in a homogeneous material, within the precision of the analysis. So, it can be inferred from virtually all of the data given above that the structure of Genesis diamond-like carbon collectors varies laterally on a 250 × 250 µm scale (the scale of sputtering). Moreover, from the SRIM models coupled with the profile shape factors (Fig. 3) it can be inferred that the effective density of each column of DLC sputtered varies laterally on the 150 × 150 µm scale (the scale of the ion collection). The question is: what we can learn about the Genesis DLC from this data? We propose that, when the ion yields change as a function of S/nA (Fig. 4) and not as a simple function of density (as modeled in Fig. 3), then we may use the SIMS data to infer information on electrical, compositional and bonding properties of the DLC and, perhaps, changes due to SW exposure. In addition, we can estimate variations in RSF (cf., “Analysis by secondary ion mass spectroscopy” in Background) due to these variations so that the quantification of SW in DoS collectors can be both precise and accurate.

### The ^12^C_2_^+^/^12^C^+^ ratio

We note that the calculation of RSF only uses one matrix ion but this study collected two: ^12^C^+^ and ^12^C_2_^+^. In a homogeneous material, it theoretically shouldn’t matter which matrix ion is used for reference. Practically, normalizing to ^12^C^+^ would be more precise than normalizing to ^12^C_2_^+^, as the sputter yield (cps per nA) is significantly higher. However, in previous SIMS sessions analyzing DLC, we had observed lateral deviations in the sputter yields of matrix ions per nA, including occasional “dead spots” (areas of extremely low cps) [26]. We hypothesized that lateral variations in electrical conductivity [5] caused areas of the DLC to be ineffective at conducting away the charge from the incoming ion beam: not enough to cause an electrical discharge, but causing some areas of the sample to be at a higher voltage than others. Since the sputter yield of ^12^C^+^ and ^12^C_2_^+^ ions differs in their energy spectrum, we suspected that variations in the local effective sample voltage might manifest as variations in the measured ^12^C_2_^+^/^12^C^+^ ratio. To test this hypothesis, we collected both matrix ions.

#### Standard Profile 4

**Figure 11:**
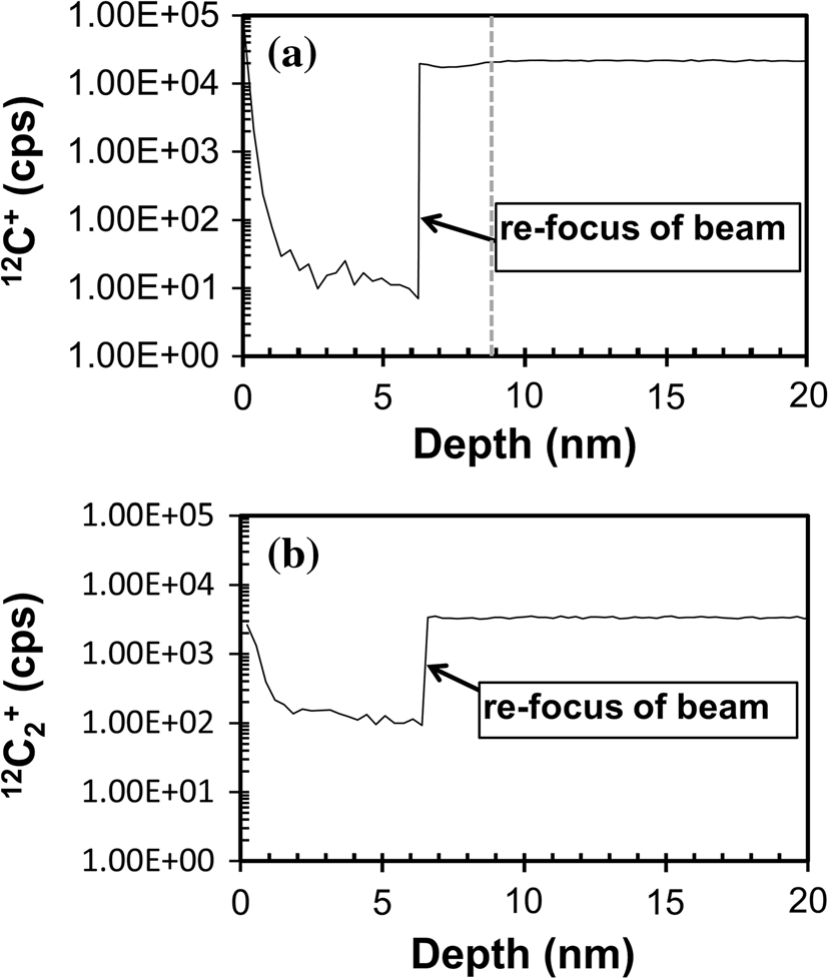
**a, b** Raw Intensity of ^12^C^+^ ions **a** and ^12^C_2_^+^ **b** versus depth for the first 20 nm of Profile 4 with depth if a constant sputtering rate throughout is assumed instead of the differential sputtering rate assumed in Figs. 5 and 6. This profile was begun using a round primary beam “spot” with uniform intensity, and then refocused to a tight “spot” having high intensity but which was not quite round. The location on the sample and the nominal raster size were not changed. Note that, under the round but slightly less concentrated beam the ^12^C starts roughly at the intensity of the refocused beam, but then drops three orders of magnitude (**a**). The ^12^C_2_^+^ also starts roughly at the intensity of the refocused beam and then drops, but <2 orders of magnitude (**b**). Initial signal is regained by both after refocusing. *Dashed vertical line* in **a** marks ~2.5 nm after the refocus, the depth sputtering appears to stabilize (vs. ~2.8 nm below the surface in Fig. 5). Very low carbon counts as in **a** can plausibly be equated with no sputtering; this can happen if the area being sputtered either deflects the beam or changes its effective impact energy by building up charge (cf., Online Reference)

Standard Profile 4 (the nominally 2.85 gm cm^−3^ filled circle in Fig. 3) was unique analytically in that the depth profile was done in two steps. First, the primary beam was focused to be a round, uniform intensity, spot (~30 µm in diameter). Then, after 307 s of analysis time (the first 2% = <7.0 nm of the profile), the primary beam was refocused to a “point” beam (~10 to ~20 µm in diameter, as per the other profiles) and then the depth profile was continued. The effect of the change in primary beam refocus (including minor beam realignment and geometry change which gave a higher beam current density) is given in Fig. 11 (note the logarithmic scale of Fig. 11 versus the linear scales of Figs. 5a, b, 6a, b). Under the slightly more diffuse beam, the ^12^C^+^ intensity had dropped more than three orders of magnitude (from ~22000 to ~7 cps) in those 307 s, while the ^12^C_2_^+^ intensity dropped <2 orders of magnitude. Counts were restored after the refocus.

It is always possible that an anomalous SIMS analysis is due to operator error; however, it is also plausible that the drop in ^12^C^+^ counts in the first seconds of Profile 4 indicated that Profile 4 was in one of the occasionally observed DLC “dead spots.” If, as we believe, the anomaly in Profile 4 was caused by the DLC and not an error setting up the SIMS (discussion in SOM), then this analysis may have been run in a low-conductivity zone: an area of anomalously high *sp* ^3^/*sp* ^2^ concentration which caused some charging of the sample (i.e., a locally high sample voltage), accompanied by significant deflection and/or local deceleration of the primary ion beam. If we then assume the lower limit for sputtering in those first 307 s based upon the precipitous drop in C counts (i.e., no sputtering), then we calculate a new sputtering rate and different shape parameters for the remaining 98% of this profile (open circle in Fig. 3; Profile 4b in Table 1). Note that the open circle calculated for this model is offset from the line representing #906 Nuclear Grade Graphite, and that the offset is likely exaggerated, as there was almost certainly some sputtering in the initial 307 s. This offset may be consistent with a dense surface layer over a less-dense substrate (past the peak); however, SRIM modeling suggested that a simple gradient density is unlikely to be responsible. SRIM modeling did imply that the location of the open circle is consistent with either an increased concentration of silicon or a decrease in the “compound correction,” the empirical factor added to SRIM 2008 to account for non-ideal changes in the bonding energies of a material. (Note: no correction for a truly tetrahedral, covalently bonded carbon matrix, e.g., diamond, is cataloged in SRIM). Profile 4 had lowest matrix counting rates and the highest ^12^C_2_^+^/^12^C^+^ ratio for the standard analyses even after the refocus (Table 1). In addition, there were crystals (inferred to be diamond) observable by SEM in portions of the adjacent analysis pits (Fig. 7) and these adjacent analyses show periodic fluctuations in the ^12^C_2_^+^ and ^12^C^+^ counts in Figs. 6b, a and 5b, a.

#### Meanings of the ^12^C_2_^+^/^12^C^+^ ratio

The ^12^C_2_^+^/^12^C^+^ ratio (the normalized matrix molecular ion yield) seems to be a useful tool for understanding the properties of DLC films for several reasons. *First*, the relative ion yields of ^12^C^+^ and C_2_^+^ appear to vary as a direct function of the local bonding structure of the DLC film. Higher strength carbon bonds (*sp* ^3^, especially those which are tetrahedrally coordinated) may give a higher percentage of ^12^C_2_^+^ ions than do other carbon bonds. This may simply be because there are more tightly bonded C-atoms; however, it may be due to very localized charging of diamond crystallites which might have reduced the impact energy of the primary beam sputtering the crystals (only) to levels equivalent to static mode under our SIMS conditions (cf., Fig. 7c and SOM) which are the extreme case of a concentration of tetrahedrally coordinated *sp* ^3^ bonds and an extremely good electrical insulator. So, in DLC bonding structure and electrical conductivity are not independent.

*Second,* the ^12^C_2_^+^/^12^C^+^ ratio can be a function of the electronic properties of the DLC because of the difference in their energy spectra (Fig. 11). We know that the electrical conductivity in a tetrahedrally coordinated carbon film is controlled by a “hopping mechanism” [5] which depends upon the size, interconnectivity, and relative proportions of *sp* ^3^- and sp^2^-bonded areas. Large variations in resistivity have been observed for areas ~50–400 µm in diameter within single samples of this type of DLC [5]. So, electrical conduction in this type of film is more effective in some areas than others and adjacent columns of material may vary.

Consider an isolated column of film: if the column is a glassy or graphitic carbon, then the electrical conductivity is high, and the current from the incoming primary ion beam will be dissipated quickly. In contrast, if the column is truly diamond, then it will be an excellent electrical insulator, so current from the incoming primary ion beam will be retained until the full capacitance of the diamond is reached and the system discharges (electrical arc). However, tetrahedrally coordinated carbon film is, by definition, somewhere between those two extremes of glassy (or graphitic) carbon and diamond (cf., Fig. 1). We might expect some areas to act as an imperfect capacitor: the electric charge builds up because the current from the incoming primary ion beam is inefficiently dissipated; however, sufficient leakage current flows so that the system never suddenly arcs. The initial portion of Fig. 11 is likely a great illustration of what happens to the relative ion yields of the secondary ions ^12^C^+^ and ^12^C_2_^+^ when the local area is poorly conductive, but never reaches the point of producing an electrical arc. In fact, one interpretation (cf., open circle in Fig. 3) assumes that charge buildup on the initial portion of the profile was so extensive that there was not enough impact energy in the primary beam ions to cause the substantial damage needed for sputtering the bulk DLC; however, the impacting ions did cause enough damage to generate molecules from the surface (cf., Fig 11: <10 cps of ^12^C^+^ and 100^+^ cps of ^12^C_2_^+^). For the calculation of the conditions for this extreme scenario see [10] and the SOM. Another possibility at low impact energy is that the *sp* ^2^-bonded carbon is more quickly eroded than the *sp* ^3^–bonded carbon (e.g., [6]). Whichever model is used, Fig. 11 implies that if charge buildup in the DLC film during SIMS analysis, then the ^12^C_2_^+^/^12^C^+^ ratio increases.

The *third* way that the ^12^C_2_^+^/^12^C^+^ ratio may change is by adding a minor element. For example, if a significant number of carbon bonds were diluted with Si (e.g., C=C was replaced by Si=C, etc.), then the ^12^C_2_^+^/^12^C^+^ ratio might decrease due to the change in structure. Similarly, if enough H was added to affect the matrix chemically, then the ^12^C_2_^+^/^12^C^+^ ratio might go up or down, depending on the relative chemical affinity of H for the different bonds (e.g., C≡C or C=C or C–C). In addition, DLC is a semiconductor, so minor elements could, potentially, change the local electrical conductivity. However, unlike more traditional semiconductors, it takes a large concentration of dopant to change the electrical properties, to the extent that it has been suggested that the dopant is simply graphitizing the DLC [27].

#### Using ^12^C_2_^+^/^12^C^+^ to understand matrix structure in the standard

**Figure 12:**
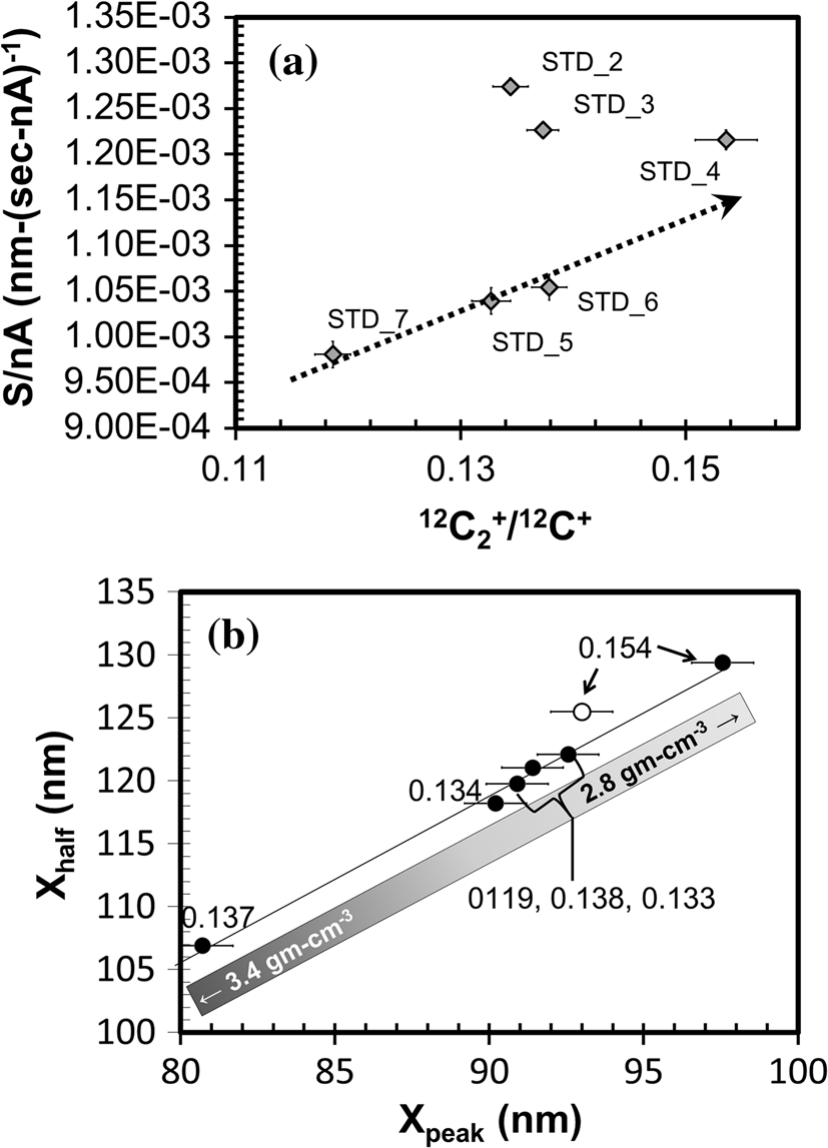
**a, b** In **a**, the plot of S/nA versus ^12^C_2_^+^/^12^C^+^ for standard implants, ^12^C_2_^+^/^12^C^+^ is the average for the entire depth profile and nA is the average of the initial and final beam current for each profile (labels = analysis). **b** plots the shape parameters for the depth profiles in **a**. *Markers and solid black line* as per Fig. 3. The two clusters defined in Fig. 4 exist in **a**, but have more internal structure: e.g., low S/nA cluster is linear (R^2^ = 0.996, *arrow*); high (nearly constant) S/nA perhaps decreases slightly with ^12^C_2_^+^/^12^C^+^. Conversely, in **b**, shape factors do not cluster. So, there may be no correlation between the clusters of Fig. 4 and SRIM-estimated density (*shaded bar*) or ^12^C_2_^+^/^12^C^+^ (*marker labels*). Note: if the SRIM-estimated density calculation for Profile 4b had been run with a lower compound correction (as might be expected for a material with a large band gap like diamond), the model density would be ~3.3. If so, model densities may be clustered (low S/nA ~ 3.0 gm cm^−3^, high S/nA ≥ ~3.1 gm cm^−3^)

Figure 12a shows that the S/nA plots still fall into two groups when using ^12^C_2_^+^/^12^C^+^ instead of the total Mg ion yield (normalized to ^12^C^+^) for the implant (i.e., Fig. 4). (Note: the matrix ion yields are controlled by a different, albeit overlapping, set of factors than the Mg ion yield—cf., [22]). The data clusters show more structure when S/nA is plotted versus the ^12^C_2_^+^ yield rather than the ^26^Mg^+^ yield, both normalized to ^12^C^+^. The variations among Profiles 2, 3, 4 (high S/nA, low ion yield cluster) are not a linear function of the effective average density, as inferred from Fig. 3. Moreover, Profile 4 has the slowest sputter rate within this group, as well as the lowest bulk density—a relationship which is counterintuitive: an otherwise homogeneous, amorphous material having a variable density should have sputtering rates inversely proportional to density. This is illustrated in Fig. 12b, in which the densities used in the SRIM calculations are highlighted by a graded bar. Profiles 5, 6, 7 (low S/nA, high ion yield cluster) are clearly linear on this plot, but appear to be an *inverse* function of density (Fig. 12b: cluster indicated by bracket)! So, the trends in Fig. 12a, b must be explained by differences in composition and/or the concentration, size, and shape of the *sp* ^3^- and *sp* ^2^-bonded areas.

##### Chemical explanation for the clusters of Fig. 4

Using SIMS and SEM on other samples of Genesis DLC, we have found that minor amounts of silicon are unevenly distributed in the film. Silicon is a common impurity in commercial graphite, even when highly refined [21]. At a low intrinsic oxygen fugacity, concentrations of Si can react with graphite to form SiC; in fact, grains of SiC ~2 µm in diameter were observed in a Genesis-flown DoS collector [18]. If Profiles 2, 3, and 4 are in an area of low silicon, then the faster sputtering rate likely reflects the enhancement of sputtering by carbon removal through the chemical oxidation of the matrix to CO, CO_2_ gas by interaction with our O_2_^+^ primary ion beam (e.g., [28, 29]). In that scenario, the slightly lower sputtering rate for Profile 4 may be due to either (1) less effective oxidation of a mostly covalently bonded area or (2) a primary ion beam with lower effective impact energy due to a locally higher sample voltage.

If Profiles 2, 3, and 4 (the high sputter rate cluster) are in an area of low Si, could Profiles 5, 6, and 7 (the low sputter rate cluster) reflect higher Si concentrations? Reduced Si reacts with O to form SiO_2_, which is a solid and, unlike CO, CO_2_ gas, must be physically sputtered away. So, residual silica might armor the carbon from oxidation, thereby slowing the sputtering. In theory, the shape, distribution, and volume of the resultant SiO_2_ could contribute to increasing the sample voltage locally, thus lowering the effective impact energy of the ion beam. However, we have routinely sputtered silicon collectors during SIMS with O_2_^+^ primary beams without difficulty [26] so “dead spots” and similar reductions in ion yield are not due to the sample voltage increasing due to the presence of SiO_2_. Instead, if the carbon matrix begins forming SiO_x_ species (an oxygen-bearing solid), then the matrix equilibrium oxygen concentration should increase, thus increasing the ion yield of Mg^+^ species [30]. In fact, the ^26^Mg/nA (total counts of ^26^Mg per average current) is 1.11 x 10^6^ cts, 1.24 x 10^6^ cts and 9.66 x 10^5^ cts for Profiles 5, 6, and 7 compared with only 4.29 x 10^5^ cts, 5.97 x 10^5^ cts and 1.30 x 10^5^ cts for Profiles 2, 3 and 4. While this trend follows that of the ^12^C^+^–normalized total counts given in Fig. 4, it should be noted that the Profiles 2, 3, and 4 have the three lowest total ^26^Mg/nA despite a relatively large variation in average ^12^C^+^ counts (Fig. 5a, b).

As an aside, we note the Mg fluctuation delineated by the filled arrow in Fig. 2b. When this feature is seen, it is seen at regular intervals and in all three Mg isotopes. So, this fluctuation represents trace Mg contamination deposited during the regular annealing steps which are a part of DLC fabrication. Perhaps it is only seen in analyses intermittently because Si needs to be present in order to raise the Mg ion yield so that it is above background.

If Profiles 5, 6, and 7 (the low sputter rate cluster) are in areas of the DLC containing significant amounts of Si, then it is possible that some of the variation in ^12^C_2_^+^/^12^C^+^ represents simple dilution of the number of bonds available to make ^12^C_2_^+^ during sputtering. Indeed, Profile 7, which has the lowest ^12^C_2_^+^/^12^C^+^, excess Si explains the shape of its non-equilibrium sputtering in Fig. 5b [22]. The ^12^C_2_^+^/^12^C^+^ variation may also trend with the *sp* ^3^/*sp* ^2^ ratio of the Si-free carbon bonds in the DlC (cf., “Meanings of the ^12^C_2_^+^/^12^C^+^ ratio” in this Discussion). That is, if the carbon film is becoming more covalently bonded with increasing ^12^C_2_^+^/^12^C^+^, then it may produce more C_2_^+^ during sputtering and/or change the local sample voltage, distorting the energy spectra for ^12^C_2_^+^ and ^12^C^+^. If so, the intersection of the trends given by the two clusters in the S/nA versus ^12^C_2_^+^/^12^C^+^ plot reflects the properties of silicon-free, highly covalent carbon (in the extreme case, diamond).

##### Structural factors in the clusters of Fig. 4

In general, compositional data collected by SIMS do not directly reflect the structure of the sample being analyzed. This fact is almost by definition: in order to collect compositional data, dynamic SIMS analysis requires steady-state sputtering for the ion yield to be fully quantifiable. During steady-state sputtering, the primary ion beam thoroughly mixes the sample before accelerating secondary ions to the mass spectrometer (e.g., [10] and “Analysis by secondary ion mass spectroscopy” in Background). However, in this study, it does seem possible to infer some structural information about the sample, probably because some of the heterogeneity measured corresponds with extremes of electrical conductivity, which itself is a function of extremes in bonding. For example, matrix ion depth profiles for the standards are given in Figs. 5 and 6 and some unusual features were inferred to be from variations in the DLC itself. Specifically, depth profiles from the high sputter rate cluster had either (1) both ^12^C^+^ cps and ^12^C_2_^+^ cps drop precipitously (10–20%) at somewhat regular intervals (~90.0–120.0 nm) (Profiles 2 and 3) or (2) a constant, exceptionally low yield for both matrix ions but a high ^12^C_2_^+^/^12^C^+^ (Profile 4). The small but precipitous drops occurring in Profiles 2 and 3 occur at approximately the same depths and are not seen in the ^26^Mg profile. Accordingly, these features are not due to instrumental fluctuations; rather, they are a feature of the DLC structure.

The regular intervals of feature in Profiles 2 and 3 strongly suggest that they reflect the DLC fabrication process. Genesis DLC films are about a micron thick, which would tear itself apart if deposited in a single step as the internal stresses in DLC may be many gigapascals (e.g., [8, 24]). Accordingly, to make Genesis DLC, a layer of ~100.0–120.0 nm was deposited and then the wafer was annealed. These steps were repeated until the entire 1 µm film was completed. This repetitive annealing seen by the DLC layers at depth reduces the average internal stress, and also increases conduction, probably through an increased graphitization [5] as well as possible growth and connection of the graphitized areas locally [8].

Our preferred explanation for the periodic swings in matrix ion count rates in Profiles 2 and 3 is that each precipitous drop in ion yield is a very thin sheet of low-conductivity material embedded in a more conductive matrix, and the periodicity is due to each sheet being associated with a single depositional layer of the DLC. It is likely that the small area of the standard from which the data for Profiles 2, 3, and 4 were collected had high local internal stresses along with a concentration of *sp* ^3^ bonds. The internal stress was relieved in part by densification within layers, probably during annealing, and—under the highest stresses—diamond was grown as part of the densification mechanism. This explanation is consistent with Fig. 7c, in which an image of the floor of Profile 3 shows a large diamond crystal surrounded by smaller diamond crystals as well as dense, linear features angled at about 120°. We note that an alternative explanation is that the impact of the primary ion beam initiated crystallization, a phenomenon occasionally observed during thinning of some DLC films [25, 31]. However, densification and crystallization during annealing most easily explain the large crystal size and the hexagonal (equilibrium) symmetry shown in Fig. 7c. In contrast, Profile 4 may have sampled a column of DLC consisting of a highly *sp* ^3^-bonded area which did not densify (cf., Fig. 12b). It is also plausible that Profile 4 sampled an area in which is more dense with a deeper peak position than predicted by using the SRIM model of #906 Nuclear Grade Graphite because of a large diamond component (either amorphous *sp* ^3^ or nanocrystalline diamond) which might dramatically change the compound correction factor in SRIM (cf., Fig. 3; J. Ziegler, personal communication).

In summary, Fig. 12b gives an apparent density scale for the DLC film calculated using the SRIM #906 Nuclear Grade Graphite compound which is based upon Fig. 3. This may be fairly accurate if the *sp* ^3^/*sp* ^2^ ratio is relatively constant on the scale of the SIMS analysis and if the average bonding of the DLC film is similar to the #906 Nuclear Grade Graphite. We note that [5] gives the nominal film density as 3.0 gm cm^−3^, which is the apparent density around which Profiles 5, 6 and 7 cluster on the #906 Nuclear Grade Graphite line. However, if the *sp* ^3^/*sp* ^2^ ratio of the DLC film is significantly inhomogeneous on the scale of the SIMS analysis, the calculated apparent density values may be considerably skewed. For example, a large diamond component may mean that approximating the DLC as #906 Nuclear Grade Graphite is inappropriate, so that the density of Profile 4 appears lower than it really is.

#### Using ^12^C_2_^+^/^12^C^+^ to understand matrix structure in the SW collector

The discussion above touches upon the fact that processes which affect the internal strain field of the DLC (or otherwise decrease the activation energy necessary for atomic rearrangements) may trigger either new crystallization or coarsening of existing texture (e.g., forming areas of nanocrystalline diamond [31]). The question is whether or not our SW long duration, low dose ion implant might be one of those triggers.

**Figure 13:**
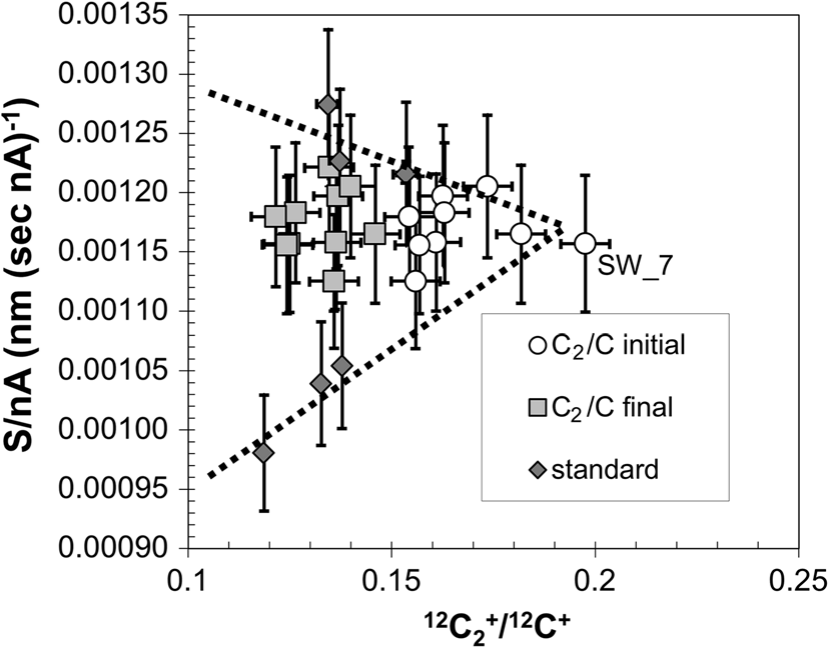
Data from Genesis–flown samples compared with standards of Fig. 12a. From Fig. 10 it can be inferred that the ^12^C_2_^+^/^12^C^+^ in the Genesis–flown DLC can change significantly with depth; accordingly both the ^12^C_2_^+^/^12^C^+^ initial (from first ~<5.0 nm) and ^12^C_2_^+^/^12^C^+^ final (at ~250 nm, after Mg analysis) are shown. *Broken line* is a visual estimate for the two trends in the standard data; the point at which they intersect may correspond to parameters for nanodiamond

Figure 13 compares the matrix of the SW collector to that of the standard by plotting ^12^C_2_^+^/^12^C^+^ final data from the SW collector versus sputtering rate (dark gray squares) on a modified version of Fig. 12a. The ^12^C_2_^+^/^12^C^+^ of the standard (open circles) is calculated for a depth of ~230.0 nm to be clearly comparable with the more shallow SW analyses. (In practice, however, the ^12^C_2_^+^/^12^C^+^ of the standard is effectively constant throughout). The dashed line forms an angle: it is drawn to approximate the trends for the two clusters of standard data discussed previously in “Chemical explanation for the clusters of Fig. 4” in this Discussion (cf., Fig. 12a). If the hypothesis for the two trends of Fig. 12a is correct, the “silicon trend” and “no silicon trend” should meet at zero silicon and the highest carbon density (e.g., nanodiamond). All of the data from the SW collector plot closer to the high sputter rate, low ion yield cluster of standard data; however, since counting times for matrix ions were short, the error on a fraction of the points are consistent with the low sputter rate, high ion yield cluster as well.

Also plotted in Fig. 13 is the S/nA versus SW ^12^C_2_^+^/^12^C^+^ initial (open diamonds). The initial ^12^C_2_^+^/^12^C^+^ are complicated to interpret for the reason that they are affected by non-equilibrium sputtering. Still, some information with respect to matrix structure and composition at the collector surface can be inferred as follows. Note that the initial SW ^12^C_2_^+^/^12^C^+^ values are all higher than the final SW ^12^C_2_^+^/^12^C^+^ values. Figure 10 shows that, for the standard, the ^12^C_2_^+^/^12^C^+^ initial is higher because the initial ^12^C_2_^+^/^12^C^+^ is controlled by lower carbon counts (cf., non-equilibrium sputtering, Figs. 5, 6). The SW collector shows the same deficit in ^12^C^+^ at the surface. However, Fig. 10 shows clearly that, in the standard, ^12^C_2_^+^ ion intensity is effectively *constant to the surface* (cf., Fig 6a, b); i.e., the initial ^12^C_2_^+^ are equal. Moreover, Fig. 10 also shows that, in contrast, the ^12^C_2_^+^ ion intensity is *higher at the surface* of the SW collector in half of the analyses (inset). The relative numbers are given in Table 3. So, the implication of Fig. 10 is that the DLC of the analytical standard is fairly uniform within each column measured, as suggested by [5]. However, the SW collector is not as uniform, having a stronger bonding and/or lower conductivity at the surface in five of the profiles. Specifically, given the arguments above, the increase in ^12^C_2_^+^ ion intensity without a decrease in ^12^C^+^ ion intensity suggests a significantly more diamond-like structure at the surface of those columns in the Solar wind collector for half of the analyses.

We should note that SW Profile 7 (labeled in Fig. 10) has an anomalously high ^12^C^+^ and ^12^C_2_^+^ ion intensity at depth, while the other SW Profiles behave in the same manner as the standard at depth.

In any case, given that the data are from only one collector fragment, we can’t know if (1) all Genesis-flown collector DLC changed during SW exposure—perhaps because of increase in mobility of atoms in the presence of the hydrogen and/or the radiation damage caused (primarily) by the hydrogen and helium; or (2) the sample of collector from this study was anomalous before flight and just happened to survive. However, these data are consistent with the possibility of some kind of “grain coarsening” in DLC which interacts directly with the SW. Support for this hypothesis comes from a possible coarsening of texture in one other Genesis SW collector, in which SEM and EDS indicated areas of micron-sized SiC crystallites in the wall of a SIMS analysis pit [20].

### How changing DLC structure affects the relative Mg ion yield

In “Standard profile 4” in this Discussion, we discussed how local concentrations of Si can increase the absolute ion yield of the implanted Mg in the analytical standard by retaining more O from the primary ion-beam in ion-beam mixed layer at the surface. However, to quantify the Mg concentration by SIMS, the relative Mg to C ratio is used, in the form of the RSF (“Analysis by secondary ion mass spectroscopy” in Background). So, while Si (and perhaps other minor components) clearly affect the Mg yield, the structure and electrical properties of the DLC matrix also directly affect the carbon yield (cf., Figs. 5, 6, 11 and Table 1). Accordingly, the value calculated for the RSF depends on the interaction of many factors. Here, we attempt to parameterize the RSF in order to learn about the structure, but also to empirically estimate appropriate RSFs for more accurate quantification of data. We note that this has also been tried in other engineered materials (cf., [32]).

**Figure 14:**
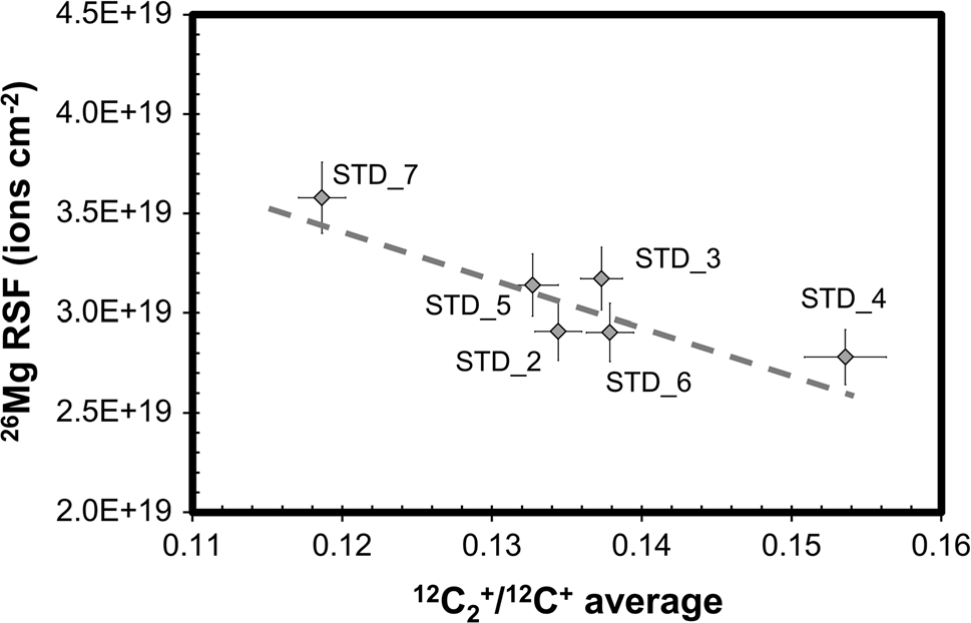
Plot of RSF versus ^12^C_2_^+^/^12^C^+^ (average for profile) for analysis of standard. Data should plot to a single point for a uniform material. Labels designate analysis; error bars are ~1σ. Unlike plots using S/nA (e.g., Figs 4, 12a) data are linear (*dashed line* for reference) and do not cluster; any material variations affecting normalized ion yield (e.g., electrical, compositional, structural) effectively cancel within counting statistics for depth Profiles 2, 3, 5, and 6

Figure 14 shows a parametrization of the RSF of ^26^Mg^+^/^12^C^+^ with respect to variations in the DLC using the ^12^C_2_^+^/^12^C^+^ ratio as a proxy for structure. Note the linear trend: when plotted versus the ^12^C_2_^+^/^12^C^+^ ratio instead of S/nA, the two ion yield clusters of Fig. 4 meld. Profile 7, the column of DLC with lowest ^12^C_2_^+^/^12^C^+^ (the most Si-rich, or the least *sp* ^3^/*sp* ^2^, or, perhaps, both) anchors the high RSF end of the trend, while Profile 4 (inferred to sample a column of DLC which is low Si and high *sp* ^3^/*sp* ^2^–perhaps even nanocrystalline diamond) anchors the low RSF end of the trend. The RSFs of the other profiles are almost a single point, within error.

This trend is not intuitive. For example, we know that Profile 7, of the “Si trend” discussed previously in this section under “Chemical explanation for the clusters of Fig. 4”, has a relatively high Mg ion yield. So, since the RSF for the standard is proportional to (total ^12^C^+^)/(total ^26^Mg^+^), if the RSF of Profile 7 is high then the total ^12^C^+^ yield per nA must be relatively higher. Similarly, Profile 4 has a low ^26^Mg^+^ ion yield per nA. Then, if the RSF is low given a low ^26^Mg^+^ yield per nA, then the total ^12^C^+^ yield per nA must also be relatively lower. Moreover, the other profiles have a similar, intermediate RSF, despite the clear separation of Profiles 5 and 6 from Profiles 2 and 3 when plotted with respect to sputtering rate (Figs. 4, 12) which likely reflects Si-content.

**Figure 15:**
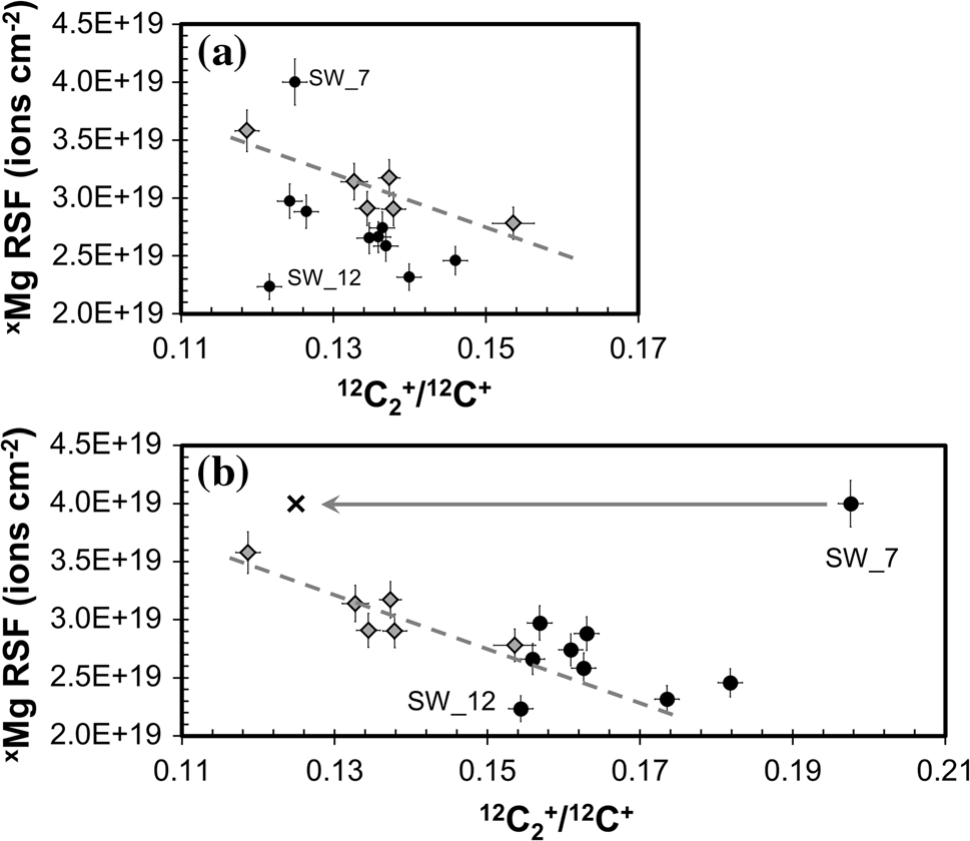
**a, b** Comparison of data from standard with data (*diamonds*) from SW collector (*filled circles*) for plot of RSF versus ^12^C_2_^+^/^12^C^+^ where **a** and **b** use ^12^C_2_^+^/^12^C^+^ final and initial, respectively. RSF for SW assumes a SW ^24^Mg fluence of 1.31 × 10^12^. Reference line from Fig. 14 is given in **a, b** for comparison. Errors are ~1σ. *Arrow* in **b** points from initial to final ^12^C_2_^+^/^12^C^+^ of SW profile 7, which has highly variable ^12^C_2_^+^/^12^C^+^

Figure 15a, b compares the SW results with the standard data of Fig. 14. This comparison was done by assuming a SW 24 Mg value as determined from analyses of silicon SW collectors (in a parallel study by D. S. Burnett) and then calculating RSFs for the profile based upon this nominal fluence. Again, the RSF trend is decidedly linear and parallels that of the standard analyses. The SW data are dispersed along the linear trend, with the exception of two clearly anomalous analyses: SW Profile #12 contained an embedded particulate [18]; SW Profile #7 had an anomalous change in ^12^C_2_^+^/^12^C^+^ with depth (cf., Table 3 and Fig. 10).

Figure 15a also indicates that the Mg RSFs are systematically lower for the SW data than that of the standard (For a homogeneous material, choice of Mg species should not affect the RSF within our statistical errors). So, if the SW fluence from the silicon analyses is correct, the relative ion yield of total ^24^Mg/^12^C from the space-exposed DLC collector is higher than that of the standard with the same nominal structure. Since SW ^24^Mg is implanted closer to the surface than the ^26^Mg implanted standard, it is possible that the surface of the film has more control on the SW implant than on the analytical standard. Accordingly, Fig. 15b plots the same SW data using the *initial* ^12^C_2_^+^/^12^C^+^ values instead of the equilibrium, final values. The linearity remains similar, but the SW RSFs are now *higher* than that expected from the standards. In fact, work in progress by D. S. Burnett uses RSFs from a best fit line to standard data (RSF vs. the ^12^C_2_^+^/^12^C^+^) to calculate SW fluences for our Genesis sample, DOS 20732–2. This test showed that (1) using initial values from SW profiles to calculate SW Mg fluences gives results strikingly divergent from values measured in Si collectors, but (2) using the final ^12^C_2_^+^/^12^C^+^ values from SW profiles to calculate SW Mg fluences give results close to that of SW measured in silicon, but about 8% high.

So, at present, it appears that the SW collector has a consistently offset ^x^Mg/^12^C^+^ ion yield (where x is interchangeably 24 or 26) from the standard. This resultant offset in RSF is corrected using the surface ^12^C_2_^+^/^12^C^+^ values of the SW collector. Perhaps when looking at the low RSF, we are seeing the effect of H on the ion yields. If so, this would be occurring in spite of the fact that neither the SW H or SW He peaks overlap the (deeper) peak of the SW Mg. As an alternative, we may be seeing the effect of radiation damage on the structure of the film which must be caused primarily by the abundant SW H and SW He. Since the ^12^C_2_^+^/^12^C^+^ values reflect both the local bonding structure and the conductivity of the bulk, it could be that the effect of radiation damage is to make conductive paths through a coarser structure or to graphitize portions of the matrix without affecting the most diamond-like bonds. Accordingly, more work is needed to determine whether it is the structure or composition of the solar wind collector affecting the relative ion yield of the DLC film during SIMS analysis.

## Summary with conclusions

Ion implants were shown to be very useful for understanding the microstructure of amorphous, tetrahedrally coordinated, diamond-like carbon films. The area sampled was 200 ± 50 µm, and the depth resolution was excellent: transient sputtering was always <4.0 nm. The evidence strongly suggests that angstrom level resolution is achievable near these SIMS conditions because of the low impact energy per nucleon of the O_2_^+^ molecule and the large band gap of diamond, combined with the tendency of the most diamond-like areas to charge under the primary ion beam, which decreases the nominal impact energy (cf., “Standard profile 4” in the Discussion and SOM). Shape parameters from the SIMS depth profiles of the implant standard (e.g., implant peak depth; the depth greater than the peak where the implant drops to 1/2 the counts) can be used to understand the structure within a column of matrix material. Models were created using the SRIM program. First, for a single implant into a material with the same composition and bonding structure, models were run for different densities. Then the set of densities were repeatedly rerun with different inputs for composition and bonding. Then, the shape parameters derived from the SIMS depth profiles were compared with the model results for given density, composition and bonding parameters.

The parameters S/nA and ^12^C_2_^+^/^12^C^+^ were used to infer that the primary factor affecting the sputtering rate of our DLC under an O_2_^+^ ion beam was the relative silicon content: if no silicon was present, the O formed only volatile oxides and the sample etched rapidly, but when silicon was present, the O reacted to form SiO_x_, a solid, which slowed the sputtering. Although Si was a controlling factor, sputtering rate was also affected by bonding (*sp* ^3^/*sp* ^2^ ratio) and electrical conductivity (*sp* ^3^/*sp* ^2^ ratio and the volume and interconnectivity of conductive paths) which are loosely parametrized by the measured ^12^C_2_^+^/^12^C^+^ ratio. For a commercial implant into DLC, ^12^C_2_^+^/^12^C^+^ can be monitored throughout the depth measurement, as well as laterally across a sample when multiple SIMS profiles are made.

The RSFs can be parametrized as a function of ^12^C_2_^+^/^12^C^+^ ratio in order to quantify SIMS data more precisely. The RSF reflects differences among the relative structure or composition of columns of material, and the properties do vary somewhat within each column. However, the factors controlling the RSF may be related to inhomogeneity at a finer scale than sputtering rate in the standard, since RSFs in the standard at intermediate ^12^C_2_^+^/^12^C^+^ values seem to cluster. Although the low-energy SW implant must have been more affected by the near-surface structure of the DLC than the higher energy commercial implant (by definition), the SW analyses were not quantified accurately when the initial ^12^C_2_^+^/^12^C^+^ values for the collector were used and RSF was determined using ^12^C_2_^+^/^12^C^+^ values from the standard; the final ^12^C_2_^+^/^12^C^+^ values for the collector seemed more accurate.

This study relates specifically to pulsed laser deposited, anhydrous tetrahedrally coordinated carbon film used in the Genesis mission [5, 9] and the effect that SW bombardment (low-energy, ~1 × 10^16^ dose, low current “H” radiation damage) has on its structure. The “preflight” DLC of the implant standard was assessed to be homogeneous through a column of material, whereas columns through the solar wind collector seemed somewhat heterogeneous, especially at the space-exposed surface. Because this was not a controlled experiment for the effects of radiation damage, there is no conclusive evidence that the anomalous structure of our specific SW collector was induced by the exposure to SW; however, at least one other SW collector fragment had observable crystallites near the SW surface [18].

The ion implant technique used here for assessing inhomogeneity and observing structural features in the DLC of the Genesis DoS wafers can, theoretically, be applied to other complex engineered materials.

## Electronic supplementary material

Below is the link to the electronic supplementary material.

Supplementary material 1 (PDF 2135 kb)

## References

[1] Burnett DS, Barraclough BL, Bennett R, Neugebauer M, Oldham LP, Sasaki CN, Sevilla D, Smith N, Stansbery E, Sweetnam D, Wiens RC (2003). The Genesis Discovery mission: return of solar matter to Earth. Spa Sci Rev.

[2] Reisenfeld DB, Wiens RC, Barraclough BL, Steinberg JT, Neugebauer M, Raines J, Zurbuchen TH (2013). Solar wind conditions and composition during the Genesis mission as measured by in situ spacecraft. Spa Sci Rev.

[3] Koeman-Shields EC, Huss GR, Ogliore RC, Jurewicz AJG, Burnett DS, Nagashiima K, Olinger CT (2016). Hydrogen fluence calculated from Genesis collectors. 47th Lunar Planet Sci Conf.

[4] Robertson J (2002). Diamond-like amorphous carbon. Mater Sci Eng, R.

[5] Sullivan JP, Friedmann TA, Dunn RG, Stechel EB, Schultz PA, Siegal MP, Missert N (1998). The electronic transport mechanism in amorphous tetrahedrally-coordinated carbon films. Mater Res Soc Symp Proc.

[6] Nakazawa H, Osozawa R, Enta Y, Suemitsu M (2010). Changes in chemical bonding of diamond-like carbon films by atomic-hydrogen exposure. Diamond Rel Mater.

[7] Tinchev SS (2012). Surface modification of diamond-like carbon films to graphene under low energy ion beam irradiation. Appl Surface Sci.

[8] Alam TM, Friedmann TA, and Jurewicz AJG. (2001) Solid state 13C MAS NMR investigations of amorphous carbon thin films‐ structural changes during annealing in thin films: preparation, characterization, and applications. Proc Amer Chem Soc,Thin Film Colloidal Sec, San Diego, CA 4/1‐5/2001, Kluwer Academic

[9] Jurewicz AJG, Burnett DS, Wiens RC, Friedmann TA, Hays CC, Hohlfelder RJ, Nishiizumi K, Stone JA, Woolum DS, Becker R, Butterworth AL, Campbell AJ, Ebihara M, Franchi IA, Heber V, Hohenberg CM, Humayun M, McKeegan KD, McNamara K, Meshik A, Pepin RO, Schlutter D, Wieler R (2003). The GENESIS solar wind collection materials. Spa Sci Rev.

[10] McPhail DS (2006). Applications of secondary ion mass spectrometry (SIMS) in materials science. J Mater Sci.

[11] Burnett DS, Jurewicz AJG, Woolum DS, Wang J, Paque JM, Nittler LR, McKeegan KD, Humayun M, Hervig R, Heber VS, Guan Y (2014). Ion implants as matrix-appropriate calibrators for geochemical ion probe analyses. Geostand Geoanal Res.

[12] Bajo KI, Olinger CT, Jurewicz AJG, Burnett DS, Sakaguchi I, Suzuki T, Itose S, Ishihara M, Uchino K, Wieler R, Yurimoto H (2015). Depth profiling analysis of solar wind helium collected in diamond-like carbon film. Geochem J.

[13] Olinger CT, Wiens RC (2010). Interpreting measured solar wind implant profiles through simulation. 41st Lunar Planet Sci Conf.

[14] Jurewicz AJG, Hervig R, Burnett DS, Wiens R, Wadhwa M, Rieck K (2009). Fractionation of Mg Isotopes Between the Sun’s Photosphere and the Solar Wind. 72nd Meteor Soc.

[15] Jurewicz AJG, Burnett DS, Woolum DS, McKeegan KD, Heber V, Guan Y, Humayun M, Hervig R (2011). Solar-wind Fe/Mg and a comparison with CI chondrites. 42nd Lunar Planet Sci Conf.

[16] Ziegler JF (2000) Stopping range of ions in matter. Online: www.srim.org. Accessed 8 July 2016

[17] Ziegler F, Ziegler MD, Biersack JP (2010). SRIM—The stopping and range of ions in matter. Nucl Inst Methods Phys Res B.

[18] Jurewicz AJG, Rieck KR, Wadhwa M, Burnett DS, Hervig R, Williams P, Guan Y, Wiens R, Huss GR (2016). New constraints on SW Mg isotopes from understanding Genesis Dos collectors, with implications. 47th Lunar Planet Sci Conf.

[19] Mayer HG (1999) Elemental analysis of graphite. Online: acs.omnibooksonline.com/data/papers/1999_644.pdf Accessed 22 Nov 2016

[20] Hladky Z, Figera M (1994). Determination of trace impurities in high-purity graphite by electrothermal atomic absorption spectrometry and inductively coupled plasma atomic emission spectrometry. J. Anal. Atomic Spectr..

[21] Canada Carbon Press Release (2016) Canada carbon achieves 99.9997% graphite purity for west block samples following nuclear graphite thermal upgrading. Online: www.canadacarbon.com/newsdetail?&newsfile=ccb_20160721.htm. Accessed 22 Nov 2016

[22] Williams P, Baker JE (1982). Implantation and ion beam mixing in thin film analysis. Nuclear Instr. Meth..

[23] Wilson RG, Stevie FA, Magee CW (1989). Secondary ion mass spectrometry: A practical handbook for depth profiling and bulk impurity analysis.

[24] Friedmann TA, Siegal MP, Tallant DR, Simpson RL, Dominguez F (1994) Residual stress and Raman spectra of laser deposited highly tetrahedral-coordinated-amorphous-carbon films, from Conference: Spring meeting of the Materials Research Society (MRS), San Francisco, CA (United States), 4–8 Apr 1994. Online: www.osti.gov/scitech/biblio/10151495. Accessed 22 Nov 2016

[25] Logothetidis S, Lioutas ChB, Gioti M (1998). Post-growth modification of amorphous carbon films under ion beam bombardment: grain size dependence on the film thickness. Diamond and Relat Mater.

[26] Jurewicz AJG, Burnett DS, Woolum DS, McKeegan KD, Guan Y, Hervig R (2008). Solar elemental abundances from genesis collectors: Fe/Mg, constraining solar-wind fip fractionation, and comparisons with CI chondrite. 39th Lunar Planet Sci Conf.

[27] Grill A (1999). Electrical and optical properties of diamond-like carbon. Thin Solid Films.

[28] Guzman de la Mata B, Dowsett MG, Twitchen D (2006). Sputter yields in diamond bombarded by ultra low energy ions. App Surf Sci.

[29] Rubshtein AP, Sh Trakhtenberg I, Yugov VA, Vladimirov AB, Plotnikov SA, Ponosov YuS (2006). Temperature effect on the formation of a relief of diamond-like carbon coatings and its modification by ion bombardment. Phy of Metals Metallography.

[30] Deline VR, Katz W, Evans CA, Williams P (1978). Mechanism of the SIMS matrix effect. Appl Phys Lett.

[31] Chu PK, Li L (2006). Characterization of amorphous and nanocrystalline carbon films. Mater Chem Phys.

[32] Gu C (2005). SIMS quantification of matrix and impurity species in III-Nitride Alloys PhD dissertation.

