# Supplementary material for: Understanding heterogeneity in Genesis diamond-like carbon film using SIMS analysis of implants
Source: J Mater Sci. 2017 Jul 5;52(19):11282–305. doi: 10.1007/s10853-017-1267-3 (PMC6979530; doi:10.1007/s10853-017-1267-3)
Supplement: Supplementary file 1 — Supplementary material 1 (PDF 2135 kb) [file 10853_2017_1267_MOESM1_ESM.pdf]

Online Resource

Re: Understanding Heterogeneity in Genesis Diamond-like Carbon Film Using SIMS Analysis of Implants (Jurewicz et. al)

This packet of supplementary material is a record of different hypotheses discussed by the authors in the form of excerpts from MS Office (Excel, Powerpoint) files as well as annotated photographs. The material is divided into five sections which are summarized in a listed.

1. A Close Look at Figure 7c

- False color image enhancing dense material and lines highlighting crystal faces on large diamond (page 3)
- Hexagonal shapes emphasize the 120 degree angles in the dense (nanodiamond?) material (page 4)

Background: the image is of the floor of a SIMS analysis pit, and only carbon was present in the EDS analysis. Accordingly, the 1/2 µm crystal was inferred to be diamond. Here, measurements of crystal faces confirm cubic lattice. In addition, light colored (high density) lineations in the matrix were interpreted as features of stress relief. Geometry highlights hexagonal geometry, consistent with the geometry of uniform 2-dimensional shrinkage; basaltic columns or mud cracks are common examples of relief of (tensile) 2D stress.

2. A Close Look at Figure 7c – Why is this beautiful crystal here? A discussion of why it isn't channeling (pages 5-9)

Background: multiple crystals were seen in the floors of SIMS pits, the most spectacular in Fig. 7c. Because it is possible for crystals to be oriented with respect to stress, it is plausible that stress-relief in these DLC films formed oriented crystals. If so, then perhaps oriented diamond crystals were excavated (not pulverized) by the SIMS primary beam because the incoming primary ions where channeled along openings in their lattice. Here, we discuss issues with this interpretation.

3. Results of calculations for lines in Figure 3: a sample spreadsheet (page 10)

Background: each line in Fig. 3 is the best fit to multiple calculations and each line uses a unique set of parameters. This sample spreadsheet shows some of the data behind Fig. 3. Note: the reason why the lines in Fig. 3 require curve fitting is that SRIM presents its results using 10 bins [16], which adds error to the calculation of the depths representing X_{peak} and X_{half}. Changing the model film thickness changes the size of the bins, and therefore may give a different apparent model depth distribution, but within error.

4. Profile 4

- Reasons why we don't think this weirdness was operator error (pages 11-13)

- Could strange intensities and molecular counts be the result of a transition to Static SIMS mode? (page 14)

Background: data produced during the SIMS depth profile changed dramatically after refocusing the primary ion beam (Fig. 11). Normally, (1) this extreme behavior would be considered operator error and (2) the analytical conditions are well into the range of dynamic SIMS [10]. These pages explore why (1) this may not have been operator error and why (2) the initial sputtering (i.e., prior to refocus) may have damaged only the surface layer of the DLC such that the analytical conditions may not have been in the dynamic range needed for depth profiling.

5. Profiles 2, 3, 4: Could inhomogenieties make very small areas charge, resulting in extra contributions of molecules?

- Physics problem on size and distribution of crystallites (page 15-16)
- Calculations for presence of very thin diamond layers in analysis region (page 16)
- How much charging would be required to enter static SIMS mode if the insulating layer is still behaving as #906 Nuclear Grade Graphite? (page 17)

Background: if Fig. 11 represented transition from static SIMS to dynamic SIMS [10] during the analytical session due to the structure of a 200 µm x 200 µm, is it possible that similar phenomenon influence SIMS analysis on a smaller scale? This section explores the possibility that, since conduction is by a hopping mechanism [5], it is possible that small areas of the DLC are not able to fully dissipate charge. If so, perhaps while they are being eroded, these insulating areas generate molecules instead of well-mixed ions (as happens during static SIMS).

A close look at figure 7c

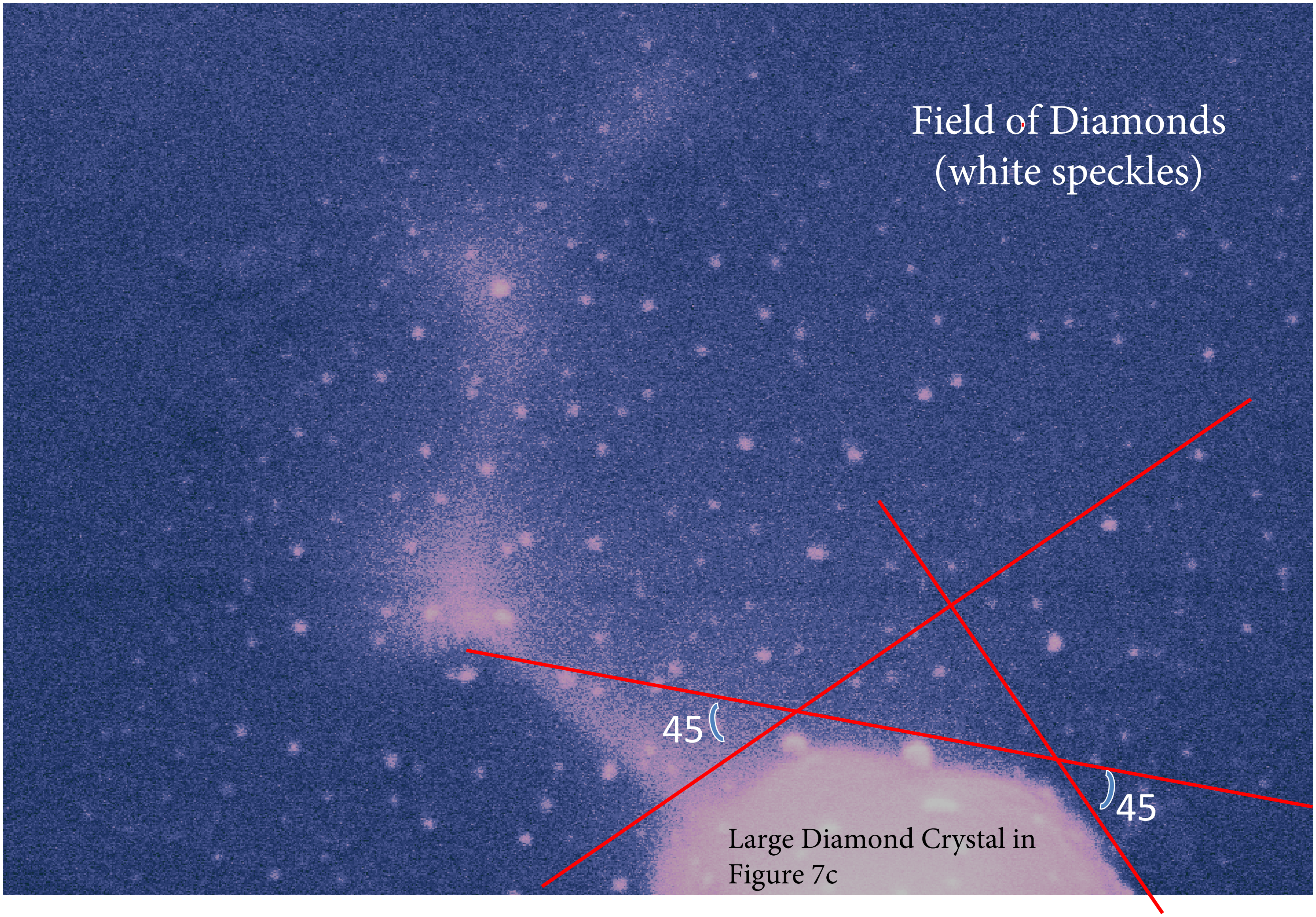

A close look at figure 7c

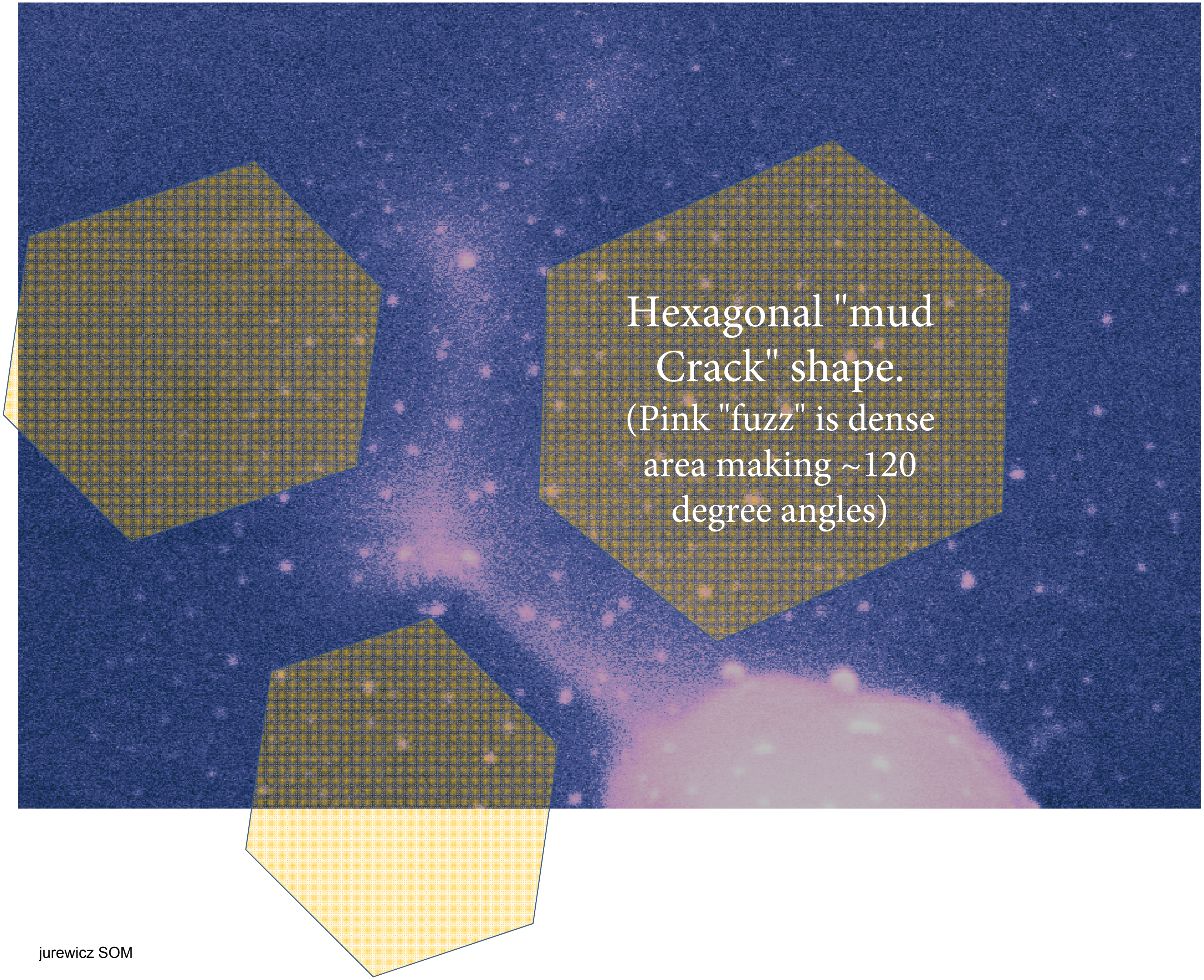

A close look at figure 7c - why is this beautiful crystal there!

Why are these corners and edges so sharp?

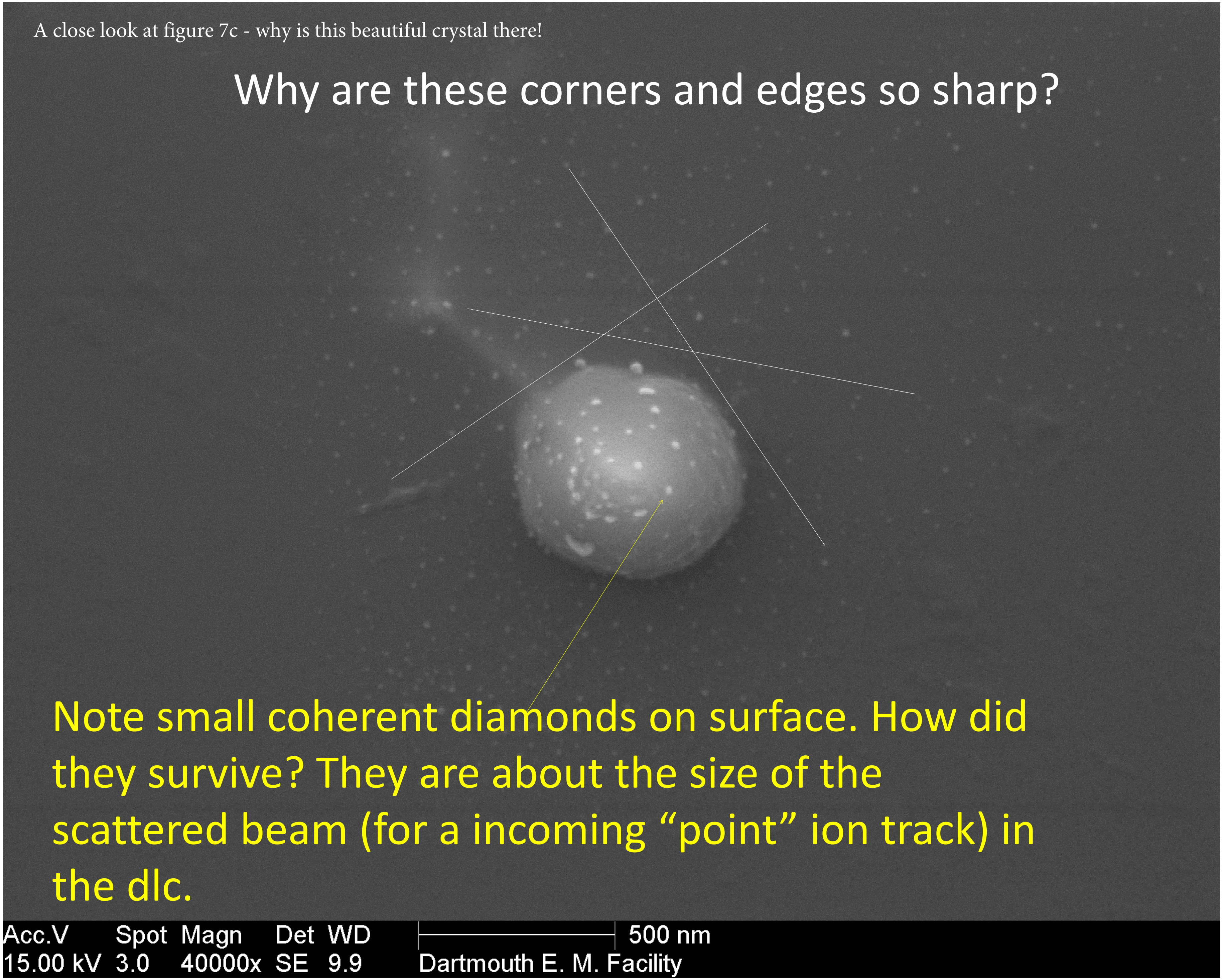

Note small coherent diamonds on surface. How did they survive? They are about the size of the scattered beam (for a incoming “point” ion track) in the dlc.

jurewicz SOM

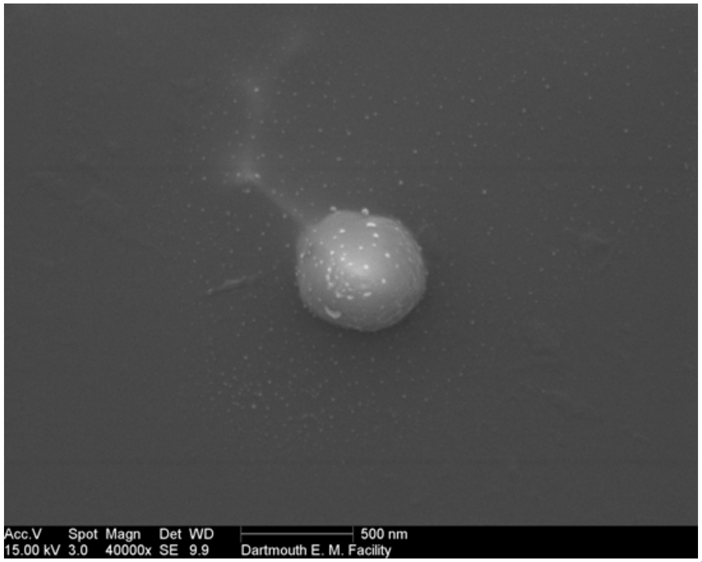

A close look at figure 7c - why is this beautiful crystal is not there because of channeling....

What We See

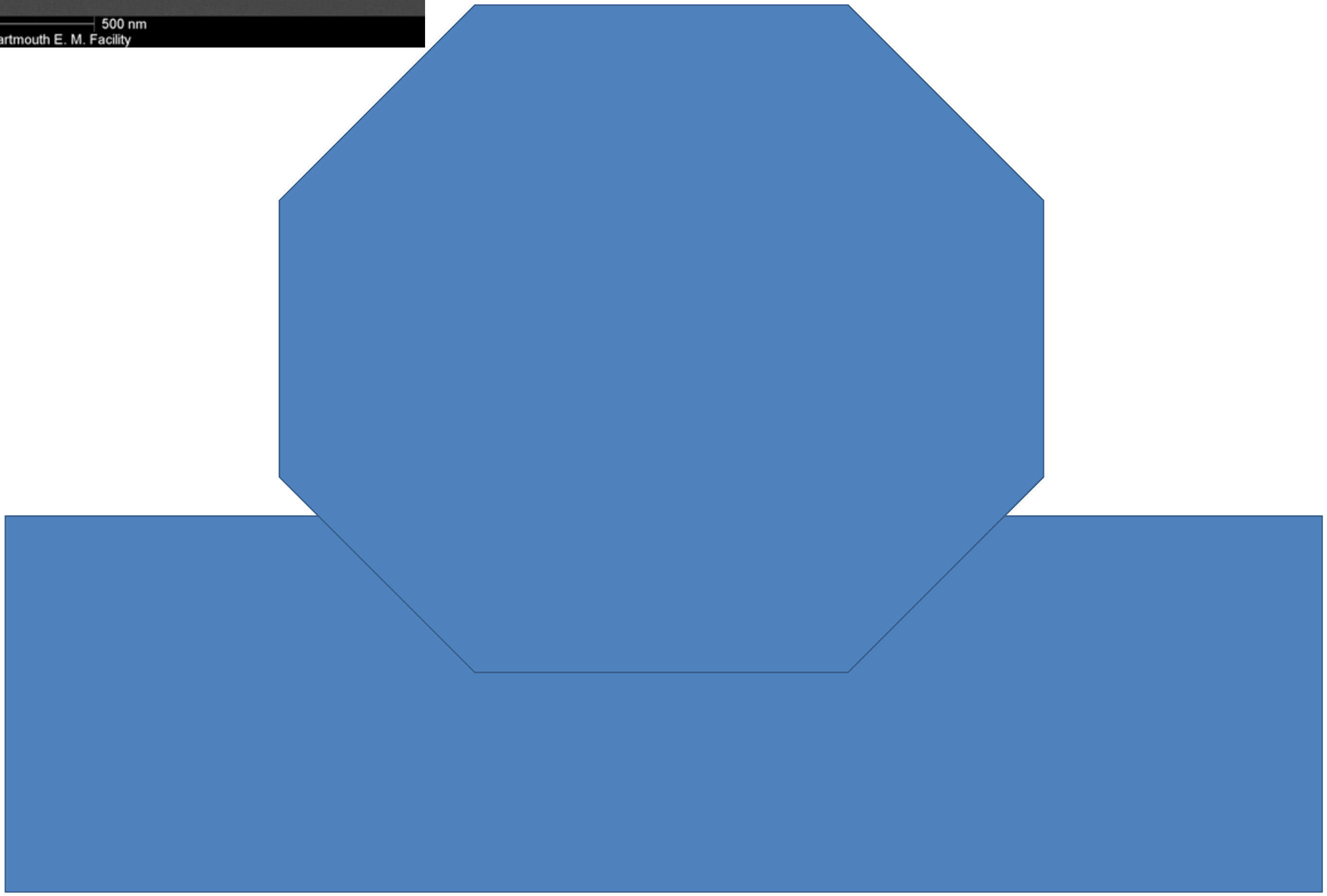

A close look at figure 7c - why is this beautiful crystal is not there because of channeling....

Model for perfect channeling:

- crystal oriented to primary beam
- no diffraction or scattering at either at surfaces of crystal or boundaries of "channels" through crystal
- ~4keV O primary

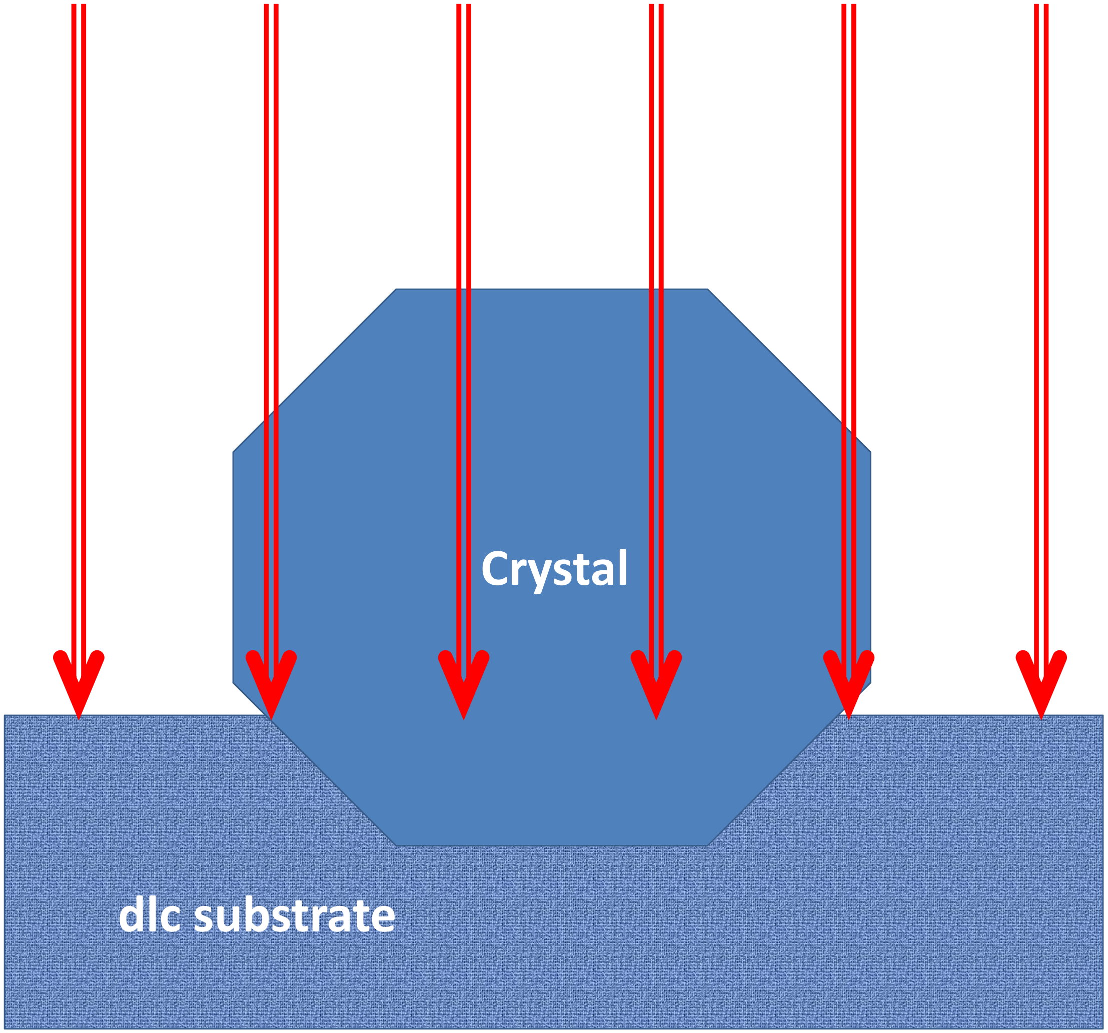

A close look at figure 7c - why is this beautiful crystal is not there because of channeling....

But diamond crystal is originally embedded in the DLC

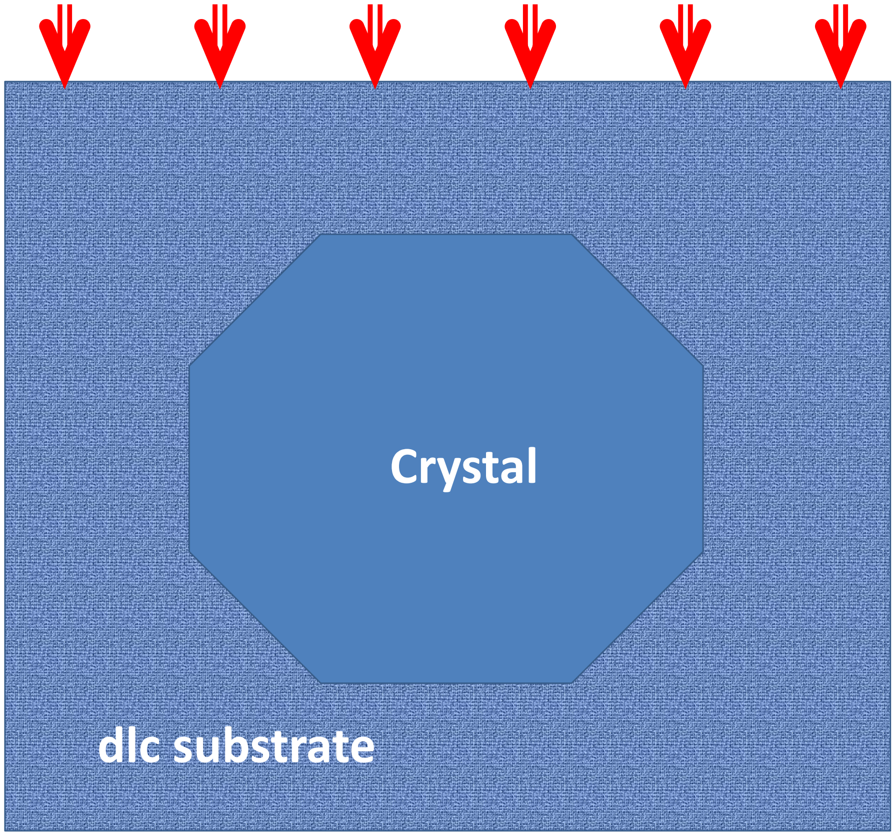

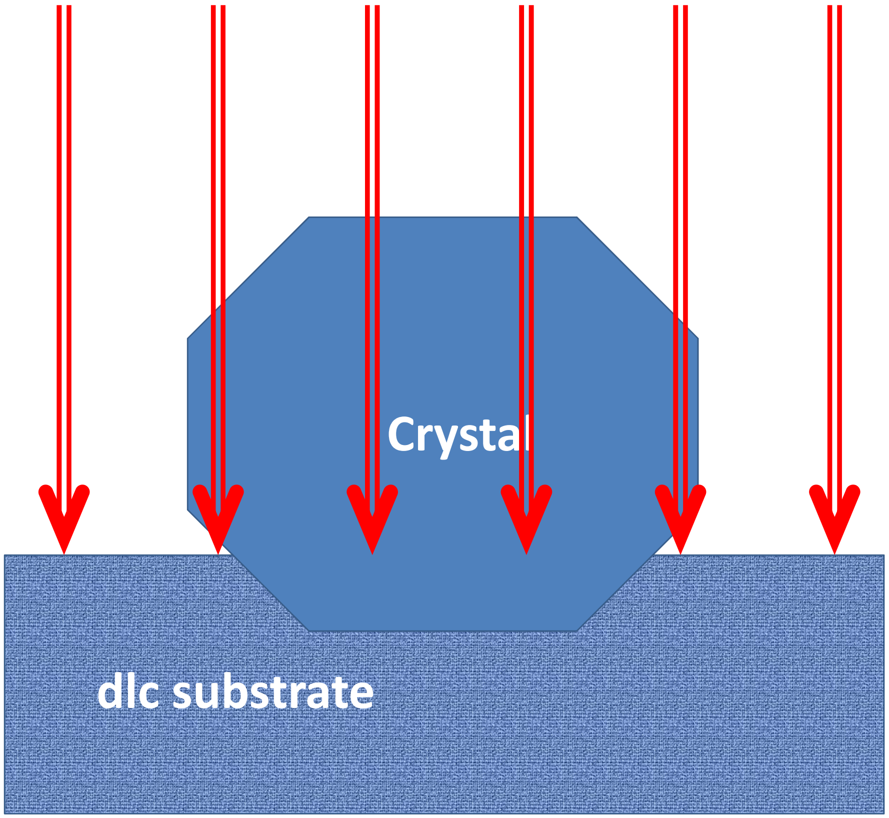

A close look at figure 7c - why is this beautiful crystal is not there because of channeling….

Assume

- Constant sputter rate = 0.266A/S (ave)
- Amount of exposed crystal =~3000A
- Time of “excavating” crystal =~3 hours
- *Every excavated surface has been exposed the 3.75keV/nucleon primary beam as scattered by collisions for approximately 6.3 minutes*

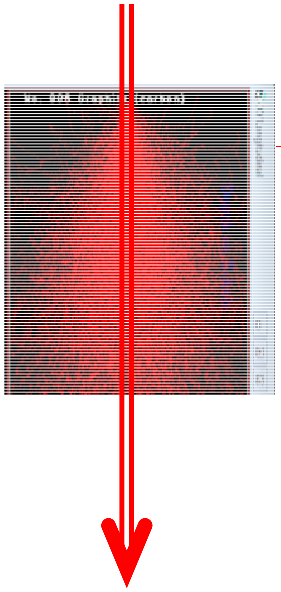

Shape of beam as penetrated DLC first 100A of surface and edge of crystal

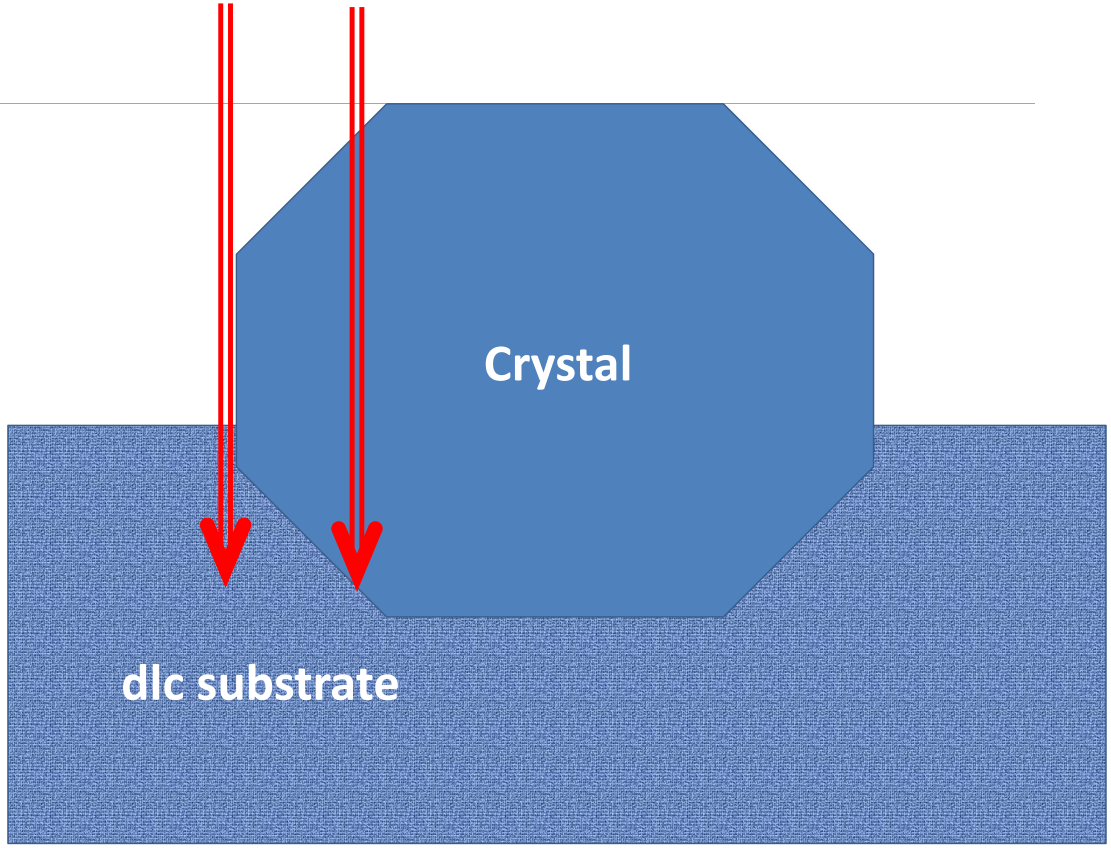

Results of calculations used for lines in Figure 3

75keV 26Mg into graphite, 7 degree tilt

file name	density	compound corr	stoich	disp	latt	surf			peak	half	ratio	
				--------damage--------------								
srim 7pt5 lattice energy graphite	2.85	1.00024	C=100%	20	7.5	2			947	1229	0.7704	
srim 11 lattice energy	3.1	1.00024	C=100%	20	11.25	2			868	1134	0.7656	
srim 7pt5 lattice energy graphite 3.1	3.1	1.00024	C=100%	20	7.5	2			868	1134	0.7657	
diamond-like maybe?	3.52	1.00024	C=100%	20	11.25	2			767	1002	0.7654	
plain c 2pt85 with graphite fudge factor	2.85	0.868400577	C=100%	28	3	7.41		graphite	965	1270	0.7604	
plain c 2pt85 withfudge factor	2.85	0.868400577	C=100%	28	3	7.41		graphite	978	1289	0.7591	
high res 3.0	3	0.868400577	C=100%	28	3	7.41		graphite	925	1210	0.7647	
12 coord 3pt1 density	3.1	0.868400577	C=100%	28	3	7.41			899	1188	0.7567	
bond 1 coord 12 3.5 g per cc	3.52	0.868400577	C=100%	28	3	7.41		graphite	797	1047	0.7612	
high res as above	3.52	0.868400577	C=100%	28	3	7.41		graphite	784	1034	0.7586	
baseline 2.85 graphite	2.85	1	C=100%	28	3	7.41			937	1214		
srim 75kev 3 pt 1 density	3.1	1	C=100%	28	3	7.41			877	1135	0.7728	
bond 1 coord 12 3.5 g per cc	3.52	1	C=100%	28	3	7.41		graphite	769	1001	0.7678	
bond 1 coord 12 2.85 g per cc	2.85	1	C=100%	28	3	7.41		graphite	948	1200.63	0.789586	
igh res	3.3	1	C=100%	28	3	7.41		graphite	805.6087	1.05E+03	0.767	
srim 75kev SiC 2pt85	2.85		SiC	20	3	2	for C	#590 SiC	1118	1565	0.7142	
carbon	3.1		85% + SiC	20	3	2		C+#590	1005	1277	0.7869	
graphite + 9 percent SiC	3.1	1	C=81.8%+SiC	28	3	7.41	for C	# 590 SiC	1032	1440	0.7170	
75 kev into SiC	3.16	1	C=50%	28	3	7.41	for C	# 590 SiC	1035	1440	0.7187	??????????
baseline 3.1 graphite +15% SiC									903.27	1194.251	0.756348	
baseline 3.3 graphite +15% SiC									848.2	1119.354	0.757759	
				best ratio								
sic structure graphite +7% Si	3	960.8	1298.361556	0.73897								
	3	948.9	1300.188562									
baseline graphite + 7%Si	3	956.5	1272.509341									
	3											
baseline graphite + 7%Si wo corr		926.0869565	1.21E+03									
sic bonded graphite no Si with corr	2.85	969	1274									
sic bonded graphite + 7%Si with corr	3	956	1269									
sic bonded graphite + 3%Si with corr	3.16	892	1171									

Profile

4**

4 no transient

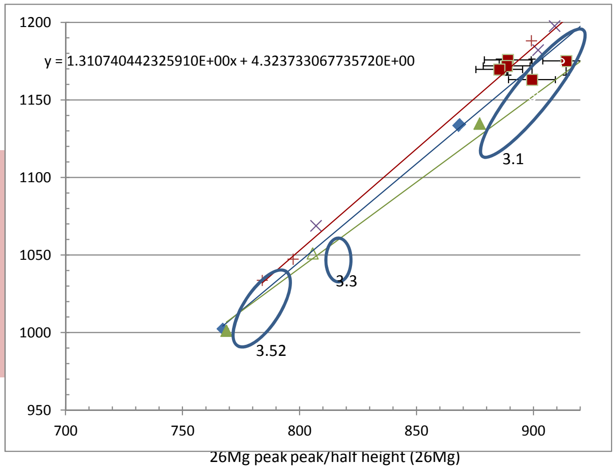

26Mg peak peak/half height (26Mg)

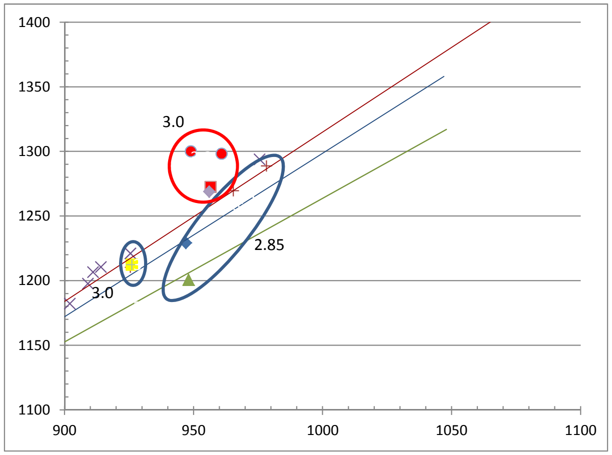

Profile 4: Reasons why we don't think that this was operator error

All data collected in first 300Å of standard Profile 4.

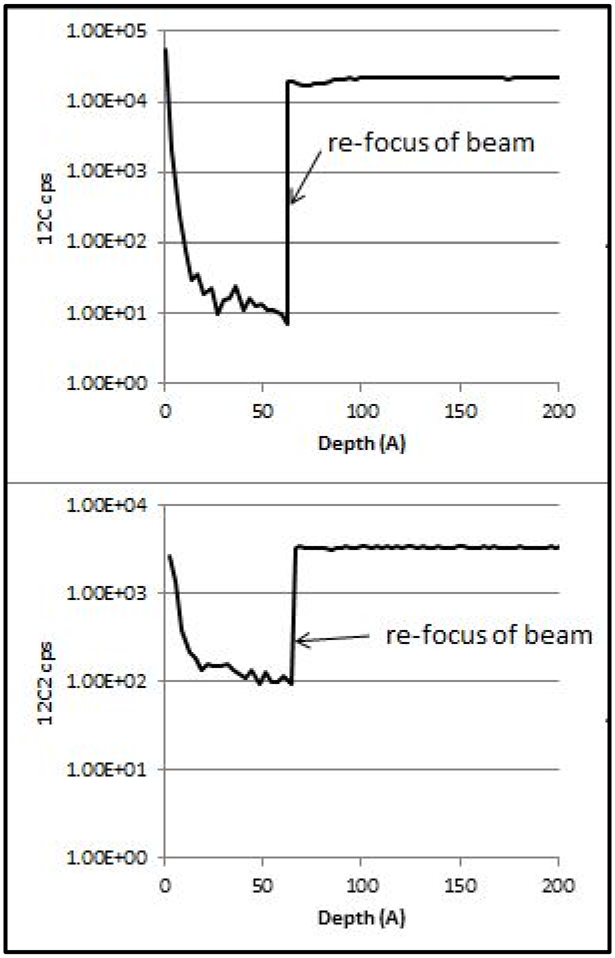

Note that counts start close the steady state value after refocus.

Rebound

(Prior to refocus) to **(stable)**

numbers are cps

ion	cps (just before refocus)	cps (~ 100Å; start new steady-state sputtering)
^{12}C	7	2.0E4
$^{12}C_2$	100	1.2E3
^{24}Mg	32	99
^{25}Mg	143	13
^{26}Mg	25	11

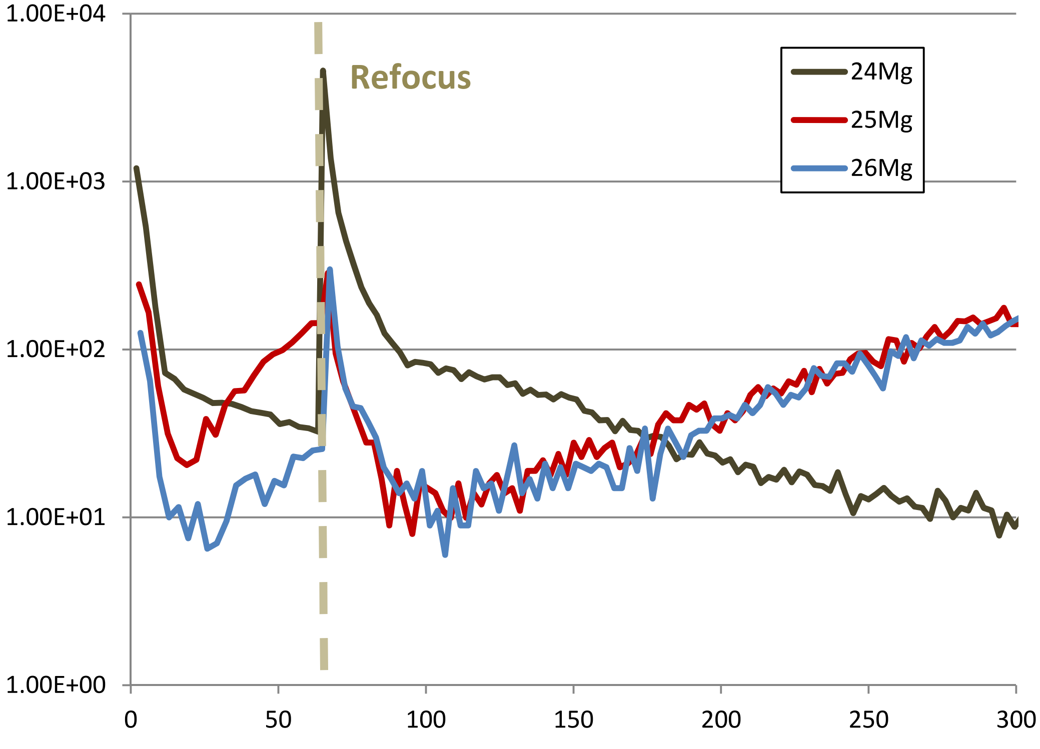

All data collected for first 300Å of standard Profile 4.

Rebound at refocus:

- ^{12}C increases 3 orders of magnitude before refocus and stays.
- $^{12}C_2$ increases less than 2 orders of magnitude. 25Mg, 26Mg drop slightly less than 1 order of magnitude and stays.
- 24Mg increases 2orders of magnitude, possibly due to surface dirt and/or deflected beam?

Full Profile of standard Profile 4 and comparison to Profiles 2, 3.

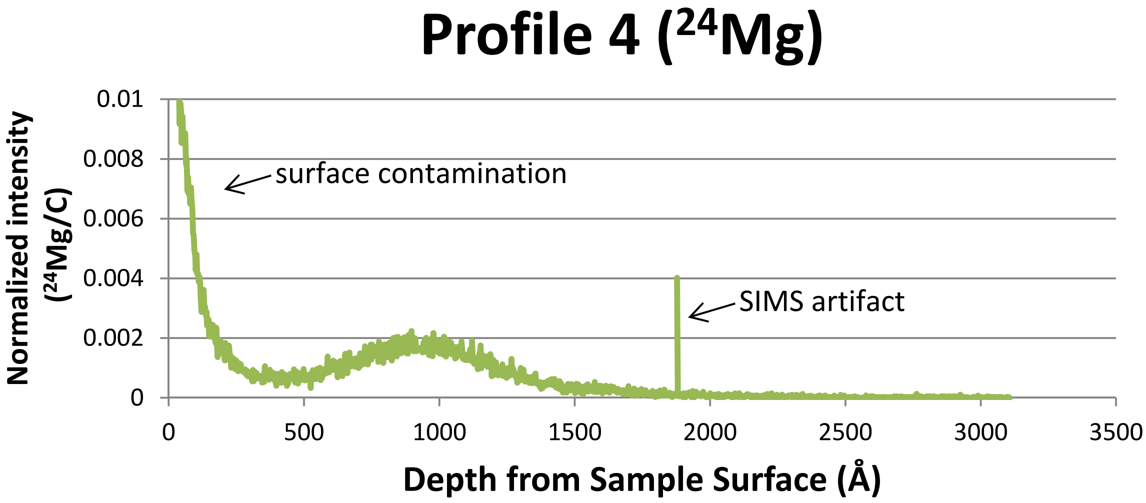

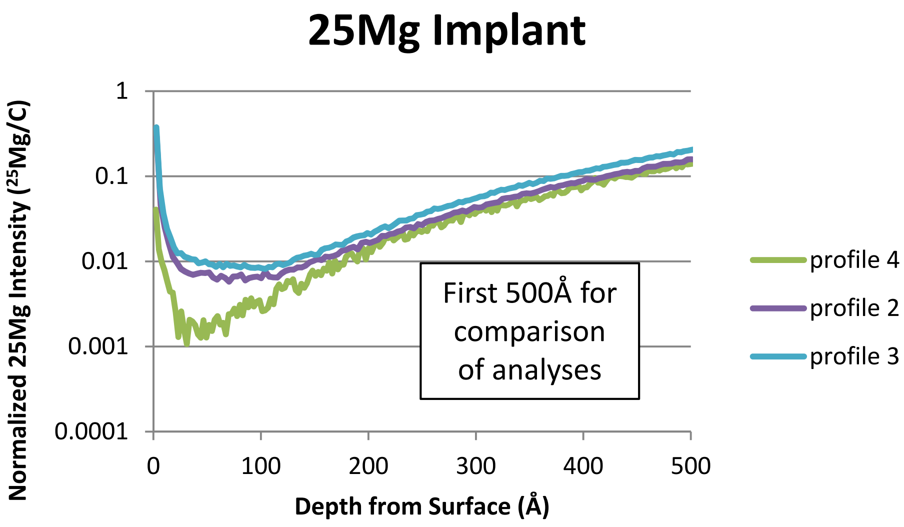

Profile 4: Reasons why we don't think that this was operator error

- Mg species profiles collected for Profile 4 looked reasonably shaped after refocus.
- Near surface contamination larger than might be hoped. (Possible deflection / misalignment / small particle? Note that beam had been realigned at refocus by senior analyst).
- Note difference in early 12C2/12C ratio in early refocused profile compared to Profile 7: in comparison, it seems anomalously high in the transient zone here, too.

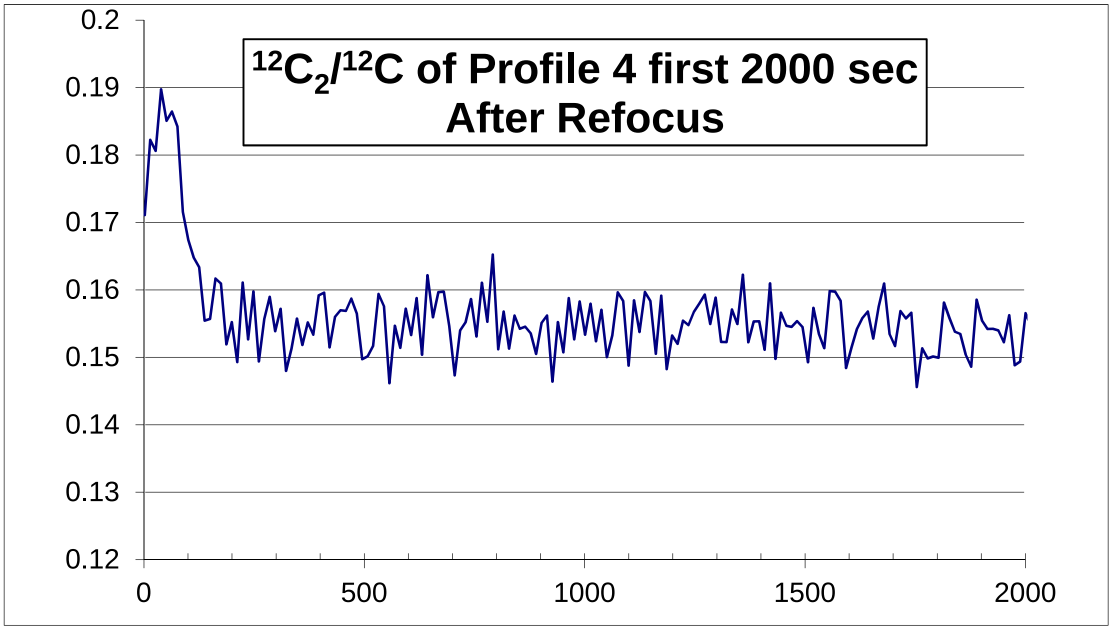

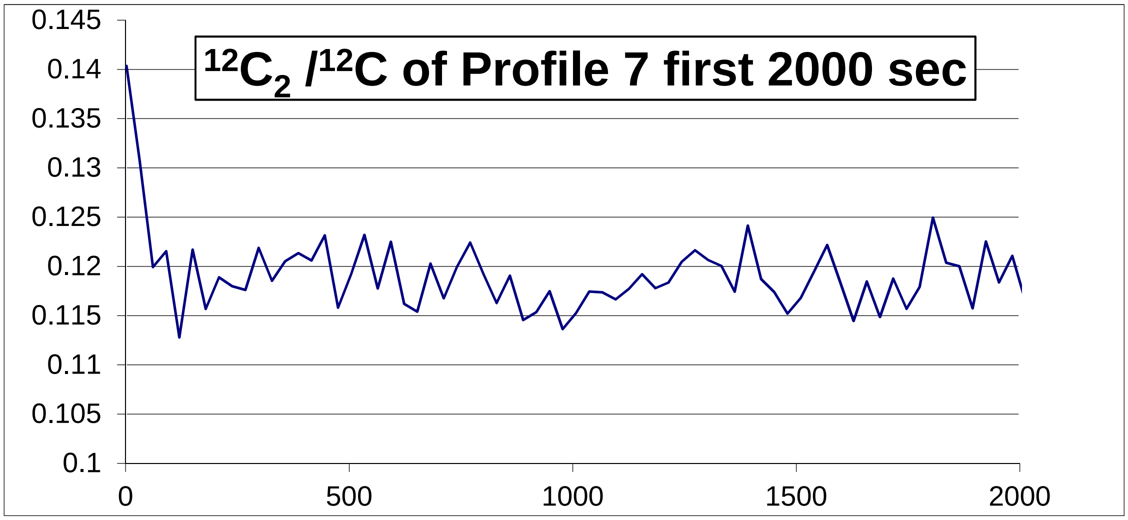

Profile 4: Could strange intensities and molecular counts be the result of a transition to Static SIMS mode?

Modeling of Profile 4
Did the weirdness in the first 307 seconds represent going from dynamic mode to static mode on the SIMS
Because the area was so diamond-like?

McPhail model of stacking spheres

if the diameter =	5	nm
diameter of the circle (nm)=	0.0000005	cm
# of circles per cm2 =	4E+12	

formula is (1cm/(diameter of circle in cm))^2

actual area of circles =	0.785398163	cm2
actual area of raster =	1	cm2

So McPhail stacking sphere model leave about 20% of area untouched by ion.

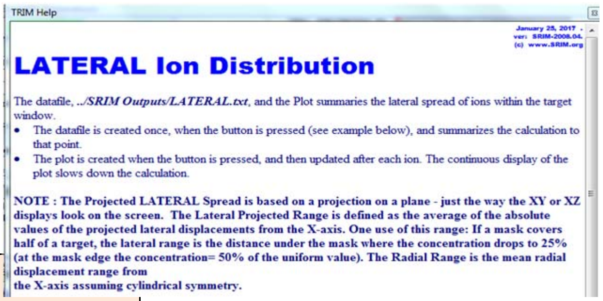
TRIM Help

January 25, 2017 - ver: SRIM-2008.04 (c) www.SRIM.org

LATERAL Ion Distribution

The datafile, *../SRIM Outputs/LATERAL.txt*, and the Plot summaries the lateral spread of ions within the target window.

- The datafile is created once, when the button is pressed (see example below), and summarizes the calculation to that point.
- The plot is created when the button is pressed, and then updated after each ion. The continuous display of the plot slows down the calculation.

NOTE : The Projected LATERAL Spread is based on a projection on a plane - just the way the XY or XZ displays look on the screen. The Lateral Projected Range is defined as the average of the absolute values of the projected lateral displacements from the X-axis. One use of this range: If a mask covers half of a target, the lateral range is the distance under the mask where the concentration drops to 25% (at the mask edge the concentration= 50% of the uniform value). The Radial Range is the mean radial displacement range from the X-axis assuming cylindrical symmetry.

SRIM of impact of Ar atom into silicon.
I chose Si because that is the major material these guys look at, so I thought it a good guess.

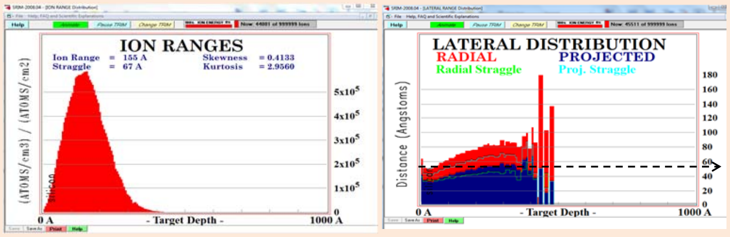

Ar @ 10keV		
range =	15.5	nm
lateral range =	5.5	nm

So, SRIM lateral range value is a slight over-estimate to what McPhail is using as a damage radius

SRIM models of O using C with compound correction of 0.6 (cf., Profile 4)
Because it is an O2+ beam, the impact for a single O nucleon is 1/2 the impact of the O2+ molecule. Nominal impact energy of molecule is 12keV.
6keV nominal for O

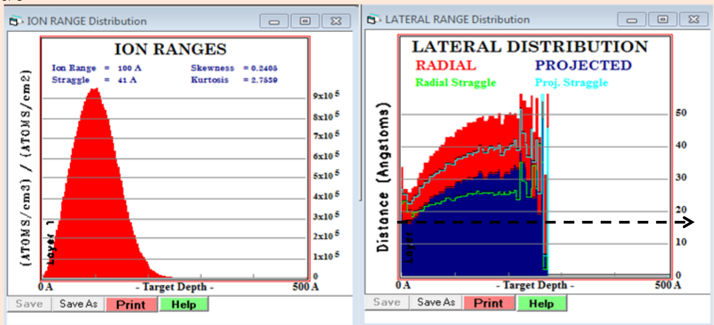

range =	10	nm
lateral range =	1.7	nm

McPhail model of stacking spheres

if the diameter =	1.7	nm
diameter of the circle (nm)=	0.00000017	cm
# of circles per cm2 =	3.4602E+13	

formula is (1 cm/(diameter of circle in cm))^2

But, the reason that the diamond-like areas need a larger compound correction is because diamond has a large band gap, which non-ideally dissipates the energy, so that less goes into the breaking of bonds and sputtering. If this is happening, the range for depth should be different as well.

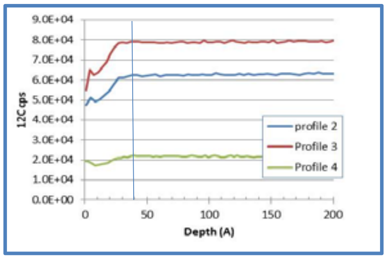

Transient depths for Profiles 2, 3 and the alternate depth reduction of Profile 4 give transient sputtering of ~0.4 nm (40 Å)
The transient depth calculated for the alternate depth reduction of Profile 4 is 10 nm

So,	calculated	empirical
range	10	4
lateral	1.7	0.68

McPhail model of stacking spheres

if the diameter =	0.68	nm
diameter of the ci	0.000000068	cm
# of circles per cm	2.16263E+14	

formula is (1 cm/(diameter of circle in cm))^2

Initial Hypothesis:

```
The ion dose is a really simple calculation. You have 2.2E-8 amps of
current (Coulombs/second). Converting from coulombs to charges, that is
1.37 E11 ions per second (and when using O2+, there are twice as many
atoms per second!). They are striking a 0.025 x 0.025 cm^2 area (actually
larger, because of the finite beam diameter!). So you can estimate a bit
less than 2E14 to perhaps 4 E14 atoms/cm^2. This is a great example of
dynamic SIMS.
```

So, we were running between ~2E14 and ~4E14.
SRIM doesn't do a great job on low energy implants, especially into non-ideal solids.
However, if we scale to what is observed, at a density of 3.3 gm/cm2 for the non-ideal DoS static SIMS starts at 2E14.
So, we were running at the very edge of static SIMS. If so, focusing and defocusing the primary beam would have changed the current
It is plausible that with the large, round beam we could have been in Static Mode SIMS,
but with the point beam, we could have been back in dynamic mode!!!!!

We note that low sputtering yields (as per diamond/large band gap) would also explain the low counts for all species in Profile 4.

Profiles 2, 3, 4: Could inhomogenieties make very small areas charge resulting in extra contributions of molecules (cf., Static SIMS mode)?

Physics problems related to sputtering and possible increased formation of molecular ions during the SIMS analysis.

1. Presence of a size distribution of insulating crystallites.

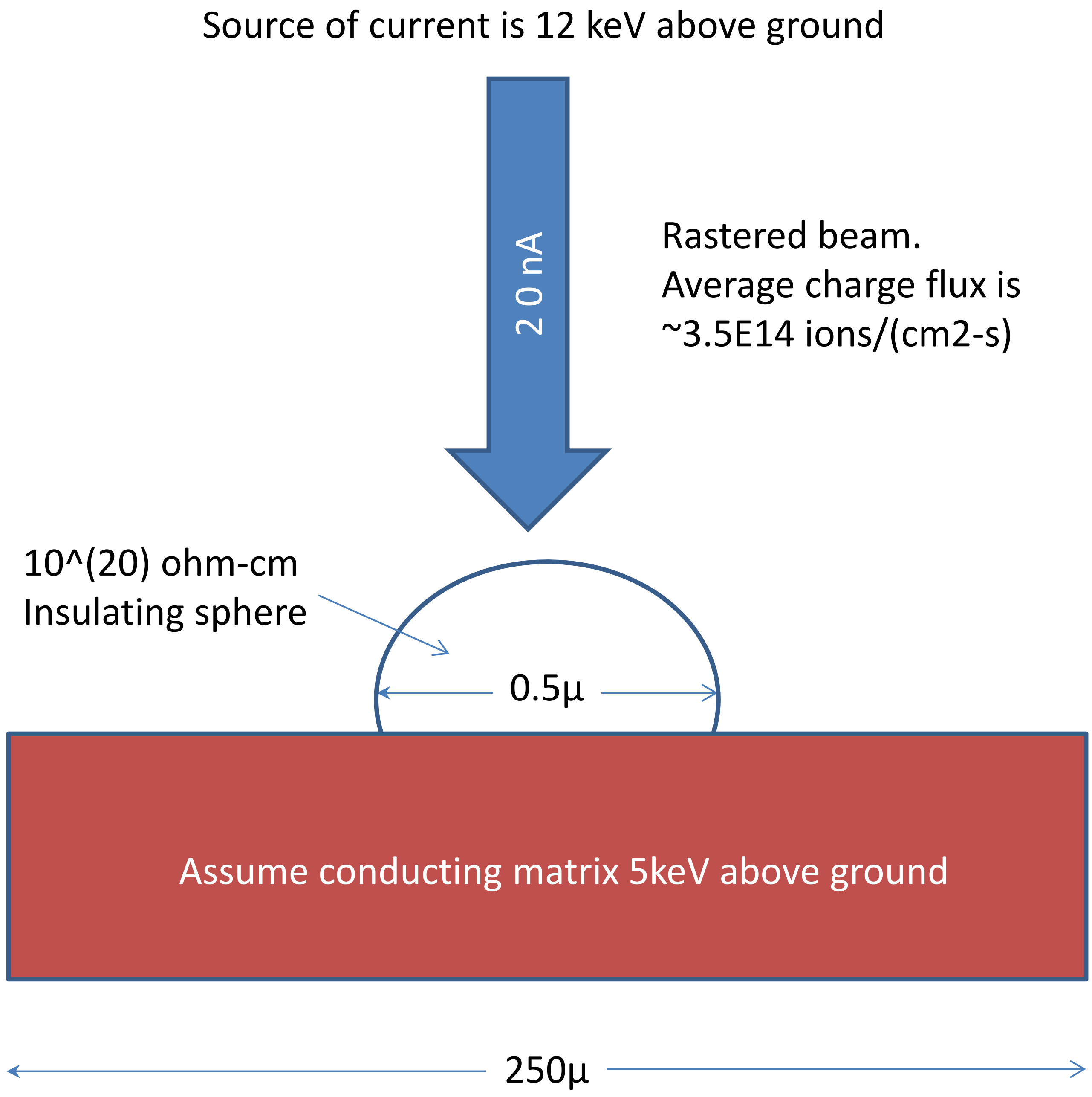

What is the likely range of charge build-up in the sphere, if any?

Profiles 2, 3, 4: Could inhomogenieties make very small areas charge resulting in extra contributions of molecules (cf., Static SIMS mode)?

- At first glance, it appears that the sphere will charge completely in a matter of seconds.
- It is likely that the amount of charging will vary (at least at first) with the net distance measured from the surface of the sphere to the conductive layer. (12kev to 0 kev on primary O ion beam).
- Primary ion beam may be deflected, or may simply be decelerated, creating little damage to the crystals.

2. Presence of insulating layers in analysis region.

mobility	1800	cm2/V-sec				
If conductivity	3.00E-07	(ohm-cm)-1		as from sullivan		
resistivity =	3.33E+06	ohm-cm				
Resistance of a cylinder =R=(resistivity)*L/A in ohm-cm						
L dlc =1 micron	1.00E-04	cm				
A sims pit =	7.29E-04	cm2				
R=	4.57E+05					
I=V/R	1.31E-05	amount of current (amps) 6 volts through dlc				
		actual = 2.2e-8 amps				
if resistivity = 1E18 ohm-meter; 1E20 ohm-cm						
R=	1.37E+19					
I=V/R	3.65E-19	Amps				
		actual = 2.2e-8 amps				

	What if a layer of diamond ro=1E120 ohm-cm?		If diamond was 1E11		if 1E14	
	L = (V/I)*(A/resistivity)		L = (V/I)*(A/resistivity)		L = (V/I)*(A/resistivity)	
	1.99E-15	cm	1.99E-06	cm	1.99E-09	
	1.99E-08	nm	19.88	nm	0.0199	
16x =	3.18E-07		318.1090909		0.318109091	

So, if a layer with an effective resistivity of 1E14 ohm-cm (low for diamond) then a 0.2 nm-thick layer would cause charging in that region.

Profiles 2, 3, 4: Could inhomogenieties make very small areas charge resulting in extra contributions of molecules (cf., Static SIMS mode)?

Estimated charging models using SRIM if the insulating layer is still behaving as 3.2 gm/cc #906 Nuclear Grade Graphite.

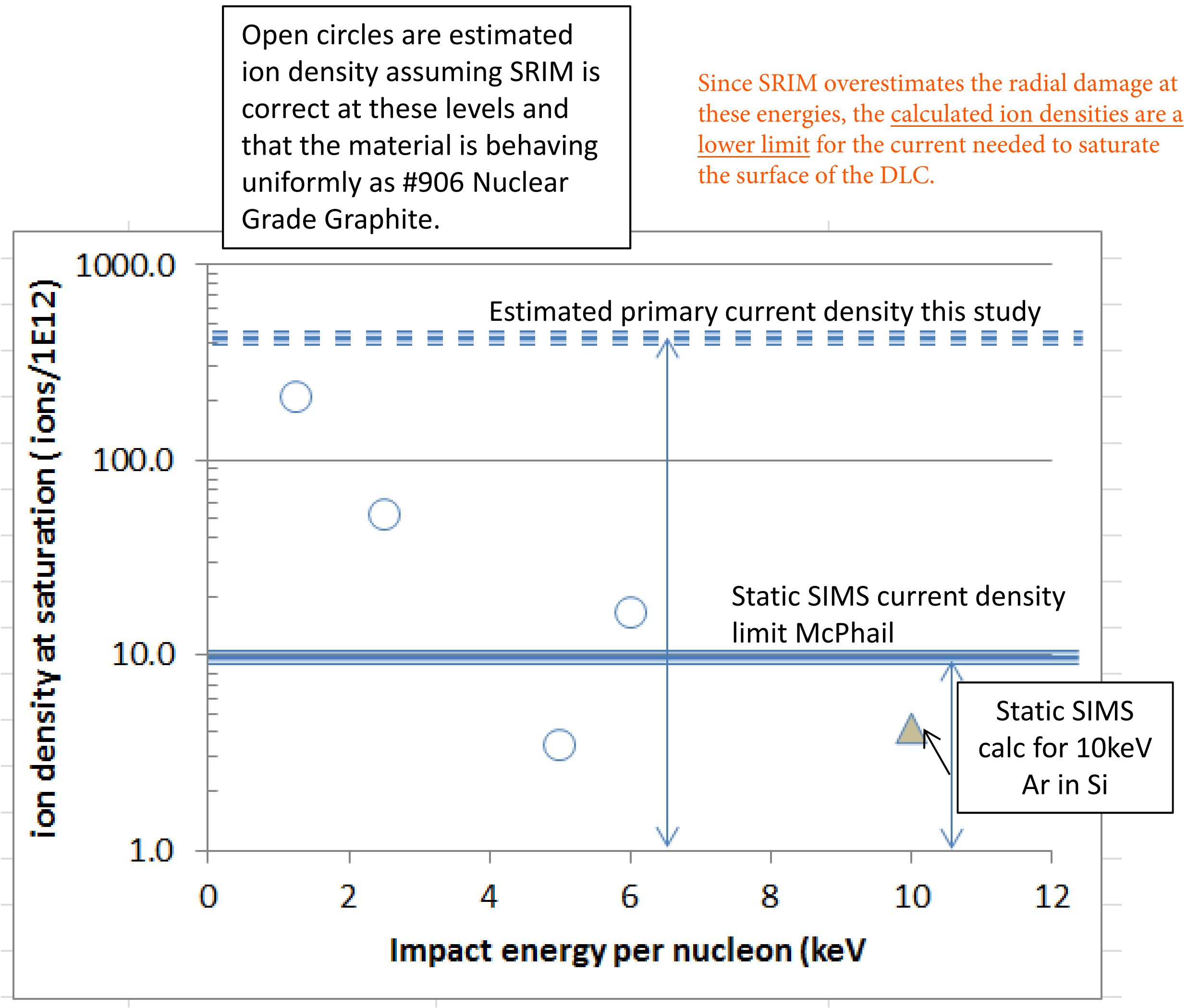

		KeV per nucleon	ion density at saturation
Si/Ar	Si/Ar	10	4.2
DLC/O2+	DLC/O	6	16.5
DLC/O2+	DLC/O	5	3.4
DLC/O2+	DLC/O	2.5	52.6
DLC/O2+	DLC/O	1.25	210.6
